# CD96 Downregulation Promotes the Immune Response of CD4 T Cells and Associates with Ankylosing Spondylitis

**DOI:** 10.1155/2022/3946754

**Published:** 2022-06-19

**Authors:** Fengqing Wu, Huan Yang, Xiao Xu, Conglin Ren, Yang Zheng, Helou Zhang, Bingbing Cai, Rui Qiu, Weifan Ren, Renfu Quan

**Affiliations:** ^1^Zhejiang Chinese Medical University, Hangzhou, China; ^2^The Affiliated Jiangnan Hospital of Zhejiang Chinese Medical University, Hangzhou, China

## Abstract

Inhibitory receptors (IRs) play an indispensable role in regulating T cell activation and expansion. This study is aimed at exploring the correlation between IRs and ankylosing spondylitis (AS). Bioinformatics analysis of two datasets (GSE25101 and GSE73754), including 68 AS cases and 36 healthy controls, demonstrated that “T cell receptor signaling pathway” was significantly enriched, and two IRs (CD112R and CD96) were downregulated in AS cases. Real-time Quantitative PCR Detecting System (qPCR) analysis confirmed the decreased expression of CD112R and CD96 in the peripheral blood of AS patients. Flow cytometry demonstrated that the frequency of CD96-positive cells among CD4 T cells in AS patients was significantly reduced and that expressed on the cells was also significantly lower than the healthy controls. In addition, the expression of CD96 was altered on human primary CD4 T cells extracted from 3 healthy volunteers and cocultured with allogeneic dendritic cells (DCs). Also, low expression of CD96 elevated the phosphorylation of ERK in CD4 T cells and increased the level of TNF-*α*, IL-23, IL-17A, IL-6, and IFN-*γ* in the cell culture supernatant. These results suggested that CD96 is crucial for the pathogenesis of AS and may be a potential target in the treatment of the disease.

## 1. Introduction

Ankylosing spondylitis (AS) is a chronic inflammatory disorder involving the spine, peripheral joints, and entheses and is a complex genetic disease of unknown etiology. Genome-wide association studies have identified >100 loci associated with AS, among which HLA-B27 and ERAP1 have the strongest association [1, 2], which speculates that AS is an autoimmune disease driven by antigen presentation-activated lymphocytes [3].

Inhibitor receptors (IRs), also known as immune checkpoint inhibitors (ICIs), are expressed on specific T cell subsets and natural killer (NK) cells. Some recent studies have shown that IRs are associated with the progression of autoimmune diseases, and abnormalities of IRs can affect T cell function and the clinical manifestations of some diseases [4]. IRs, including CTLA4, PD1, LAG3, TIM3, TIGIT, and CD96, play a certain role in the pathogenesis of autoimmune diseases [4–6]. Some studies have shown that serum CTLA-4 levels in AS patients are significantly increased and related to disease activity [7, 8]. The polymorphisms of CTLA-4 and PD-1 are also significantly correlated with AS [9, 10]. Regarding the role of IRs in autoimmunity, IRs agonists are considered a novel tool to prevent and treat autoimmune diseases [4]. T cell-targeted biologics such as abatacept also known as CTLA-4 Ig was proven to be efficient in inflammatory diseases such as psoriasis [11] and rheumatoid arthritis (RA) [12]. Unfortunately, it has not shown significant efficacy in AS [13].

Therefore, the present study is aimed at exploring the correlation between IRs and the pathogenesis of AS.

## 2. Materials and Methods

### 2.1. Subjects

Two datasets (GSE25101 and GSE73754) were downloaded from Gene Expression Omnibus (GEO). Samples of GSE25101 (16 AS and 16 healthy controls) and GSE73754 (52 AS and 20 healthy controls) were obtained from participants from Australia and Canada, respectively [14, 15]. The gene expression profiles of these samples were analyzed.

For the validation of the differentially expressed genes (DEGs), two cohorts of AS patients were investigated (Table 1). Cohort I, consisting of 19 patients and 15 gender-matched and age-matched healthy controls, was subjected to qPCR analysis. Cohort II, comprising 13 AS patients and 14 healthy controls, was evaluated by flow cytometry. All AS patients were from the Affiliated Jiangnan Hospital of Zhejiang Chinese Medical University, Hangzhou, China, and the healthy controls were recruited volunteers. Written informed consent was obtained from all the subjects before participation in the study. The present study was conducted in accordance with the Declaration of Helsinki and Good Clinical Practice guidelines, as defined by the International Conference on Harmonization, and was registered on (ChiCTR2100042212).

### 2.2. Bioinformatics Analysis

The datasets were downloaded using the “GEOquery” package of R language (version 4.0.3; http://www.r-project.org/). The probe name was converted to gene name by “affy” package and the “removeBatchEffect” function was used to eliminate the batch effect. Multiple comparisons were performed using the “sva” and “limma” packages to retrieve the differentially expressed genes (DEGs) between AS and normal controls in this integrated analysis. DEGs were identified based on the *t*-test, and the statistical significance was set to adjusted *P* value < 0.05. Kyoto Encyclopedia of Genes and Genomes (KEGG) pathway enrichment analysis was carried out using the “clusterprofiler” package to interpret the biological functions of DEGs in AS. *P* value < 0.05 was considered statistically significant. A total of 9 widely recognized IRs were collected from previously published literatures, including CTLA-4 (CD152), BTLA (CD272), LAG-3, PD-1 (PDCD1), TIGIT, TIM-3 (HAVCR2), VISTA (PD-1H), CD96, and CD112R (PVRIG) [16].

### 2.3. Reagents and Cytokines

The following flow cytometry detection antibodies were purchased from the indicated manufacturers, BD Pharmingen, San Diego, CA, USA: 7AAD, CD45 (HI30), CD3 (SP34-2), CD56 (NCAM16.2), CD8 (RPA-T8), and CD4 (SK3); BioLegend, CA, USA: CD96 (6F9), CD112R, CD80, CD83, CD86, and CD14; and Elabscience, Wuhan, China: CD40 and HLA-DR. The protein levels were detected using anti-CD96, anti-p-Akt, anti-Akt, anti-p-ERK, anti-ERK, and anti-GAPDH Abs and horseradish peroxidase- (HRP-) conjugated anti-mouse/rabbit IgG (Cell Signaling Technology, Beverly, MA, USA). DCs were stimulated using interleukin- (IL-) 4, granulocyte macrophage colony-stimulating factor (GM-CSF), IL-6, tumor necrosis factor-alpha (TNF-*α*), and IL-1*β* cytokines (PeproTech, New Jersey, USA) and prostaglandin E2 (PGE2) (Selleck, Shanghai, China). X-vivo complete medium contains X-vivo medium (Lonza, Switzerland), 10% fetal bovine serum (FBS; Gibco, Carlsbad, CA, USA), GlutaMAX (Gibco), 100 U/mL rhIL-2 (R&D, Minnesota, USA), and 100 U/mL penicillin-streptomycin solution (Gibco), and RPMI 1640 complete medium (Gibco) also consists of 10% FBS and 100 U/mL penicillin-streptomycin.

### 2.4. qPCR

Peripheral blood samples were collected into EDTA anticoagulant tubes, and peripheral blood mononuclear cells (PBMCs) were isolated by Ficoll density gradient centrifugation. Total RNA was isolated using TRIzol (Invitrogen, Carlsbad, CA, USA). The quality and purity of the isolated total RNA were tested by Nanodrop one (Thermo Fisher Scientific, Waltham, MA, USA). The RNA samples with A260/280 values between 1.8 and 2.1 were used for subsequent analyses. An equivalent of 1 *μ*g RNA was reverse transcribed into complementary DNA (cDNA) using Thermo Scientific Revert Aid First Strand cDNA Synthesis Kit (Thermo Fisher Scientific). All primers were synthesized by Beijing Liuhe Huada Gene Technology Company, Beijing, China, and the sequences are as follows:

1. *GAPDH*_F: 5′-GGAGCGAGATCCCTCCAAAAT-3′, *GAPDH*_R: 5′-GGCTGTTGTCATACTTCTCATGG-3′
2. *CD112R*_F: 5′-CATCTGCTGCGCCGACATAA-3′, *CD112R*_R: 5′-TAGTGGCATAAGGGACGTGAA-3′
3. *CD96*_F: 5′-GTCTATCATCCCCAATACGGCT-3′, *CD96*_R: 5′-CTTCCACTGACTGAACAAGACA-3′

*GAPDH* was used as an endogenous control. Then, the *ΔΔ*Ct method was employed to determine the relative difference in mRNA expression between AS patients and healthy controls [17].

### 2.5. Flow Cytometry

For flow cytometry, PBMCs were stained using monoclonal antibodies against 7AAD, CD45, CD3, CD56, CD8, CD4, CD96, and CD112R and the appropriate isotype controls for 20 min on ice in the dark; the samples were acquired in a BD LSRFortessa flow cytometer. The circling gate scheme was as follows [18]: Gate the lymphocytes according to the size and granularity (FSC and SSC). Circle the live immune cells “CD45 live” according to CD45 and 7AAD after obtaining single cells. In the gate of “CD45 live,” NK cells (CD3^−^CD56^+^) and conventional CD3 T cells (CD3^+^ CD56^−^) were sorted out. CD3 T cell (CD3^+^ CD56^−^) gate was divided into CD4 T and CD8 T cell subsets according to the expression of CD4 and CD8. The DCs were stained by antibodies against CD80, CD83, CD86, CD14, CD40, and HLA-DR and the appropriate isotype controls for 20 mins on ice in the dark; the samples were acquired using a BD C6 flow cytometer. FlowJo (v10.0) software was used for data analysis.

### 2.6. Cell Culture and Stimulation

CD4 T cells and DCs were extracted from the peripheral blood of 3 healthy volunteers. PBMCs were obtained from 40 mL peripheral blood by density gradient centrifugation, resuspended in RPMI 1640 medium, and maintained at 37°C for 2 h. The suspension cells were separated by magnetic beads (Stemcell, Canada) to obtain CD4 T cells. Dynabeads Human T-activator CD3/CD28 (Miltenyi, Germany) was added to cells at a ratio of 1 : 2. Subsequently, the CD4 T cells were cultured in a 48-well plate at a density of 2 × 10^6^ cells/mL with X-vivo complete medium. The adherent cells were stimulated with RPMI 1640 complete medium containing 1000 U/mL GM-CSF and 500 U/mL IL-4 for 6 days to obtain immature DCs, followed by stimulation with 10 ng/mL TNF-*α*, 1000 U/mL IL-6, 10 ng/mL IL-1*β*, and 1 *μ*g/mL PGE2 for 1 day to obtain mature DCs.

### 2.7. CD4 T Transfection

293T cells and lentivirus packaging plasmids (pMD2.G and pSPAX2) were donated by Professor Jinfu Wang from College of Life Sciences, Zhejiang University, Hangzhou, China. CD96-RNAi and negative control plasmids were purchased from Shanghai Jikai Gene Medical Technology Co., Ltd., Hangzhou, China. CD96-PCDH and PCDH vector plasmids were procured from Hangzhou Hein Technology Co., Ltd. Hangzhou, China (shCD96 targeting sequence: 5′-GCAATTCCACATTACTTAA-3′; shNC targeting sequence: 5′-TTCTCCGAACGTGTCACGT-3′; CD96-PCDH targeting sequence: 5′-CCTCCATAGAAGATTCTAGAATGGAGAAAAAATGGAAATA-3′). All the plasmids were packaged into lentivirus and concentrated 40-fold by centrifugation at 80000 g, at 4°C for 2 h.

After 3 days poststimulation, the CD4 T cells were added to 100 *μ*L lentivirus supernatant and 300 *μ*L X-vivo complete medium containing 10 *μ*g/mL polybrene (Sigma-Aldrich, St. Louis, MO, USA) at 37°C. After 24-h postinfection, lentivirus supernatant was replaced with fresh X-vivo complete medium containing puromycin (Sigma) at the final concentration of 1 *μ*g/mL to screen the cells with puromycin resistance. After 72 h, the cells were divided into 3 sets: one to harvest protein, one to harvest RNA, and one to be used for coculture with DCs. RNAs and proteins were extracted for knockdown efficiency verification.

### 2.8. In Vitro Coculture

Before coculture of CD4 T cells and DCs, the DCs were stimulated with 1 *μ*M ovalbumin (OVA)_322-339_ (MCE, New Jersey, USA) at 37°C for 2 h. The cells were cocultured according to the ratio of CD4 T cells to DC = 10 : 1 for 5 days [19–22]. Then, the cells were harvested for whole protein extraction for Western blotting; the supernatant was used for Luminex assay.

### 2.9. Cytokine Assays

An equivalent of 500 *μ*L of the culture supernatant was collected after centrifugation at 3000 g at 4°C for 10 min. The concentrations of TNF-*α*, IL-23, IL-17A, IL-6, and IFN-*γ* in the culture supernatants were measured using the Luminex Assay kit (LXSAHM-06, R&D Systems, Emoryville, CA, USA).

### 2.10. Western Blot

Cells were lysed in Radio Immunoprecipitation Assay (RIPA) Lysis buffer (Beyotime, Zhengzhou, China) containing protease and phosphatase inhibitors (Beyotime). Protein concentration was determined using a Pierce BCA Protein Assay Kit (Thermo Fisher Scientific). Equal amounts of protein samples were loaded and separated by 10% sodium dodecyl sulfate polyacrylamide gel electrophoresis (SDS-PAGE) and transferred to nitrocellulose membranes (Millipore, Massachusetts, USA). Subsequently, the membranes were blocked with 5% skimmed milk in Tris-buffered saline plus tween-20 (TBST) buffer (Beyotime) for 1 h and probed overnight at 4°C on the shaker with antibodies against CD96 (1 : 1000), p-Akt (1 : 1000), Akt (1 : 1000), p-ERK (1 : 1000), ERK (1 : 1000), and GAPDH (1 : 1000), respectively. Then, the membranes were treated with HRP-conjugated anti-mouse IgG or anti-rabbit IgG at room temperature for 1 h, and the immunoreactive bands were visualized using Enhanced Chemiluminescent (ECL) (Absin, Shanghai, China) and chemiluminescence imaging system (ChemiDocXRS^+^, Bio-Rad, CA, USA).

### 2.11. Statistical Analysis

Descriptive statistics included the mean, standard deviation, and/or percentages. The chi-square test was used to compare the categorical distributions, and independent sample *t*-tests were used to compare group means. *P* < 0.05 indicated a statistically significant difference. RStudio (version 4.1.0) and GraphPad Prism software (version 6.02) were used for all statistical analyses.

## 3. Results

### 3.1. T Cell Receptor Signaling Pathway Is Significantly Enriched in AS

**Figure 1:**
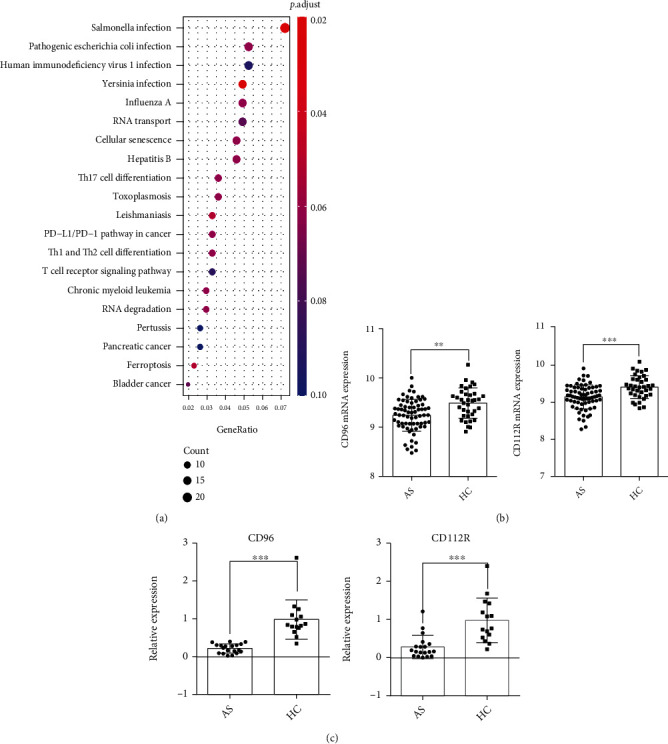
KEGG analysis and qPCR showed that T cell receptor signaling pathway and CD112R and CD96 are associated with AS. (a) Top 20 significantly enriched pathways of 642 DEGs. (b) CD112R and CD96 were significantly downregulated in AS patients through bioinformatics analysis. (c) qPCR validation of the expression of CD112R and CD96 between patients with AS and the healthy controls (^∗∗^*P* < 0.01,  ^∗∗∗^*P* < 0.001).

A total of 642 (237 upregulated and 405 downregulated) DEGs were identified with adjusted *P* value < 0.05 (Table S1). Based on the results of KEGG pathway enrichment analysis, we found that Th17 cell differentiation, PD-L1/PD-1 pathway in cancer, T cell receptor signaling pathway, and Th1 and Th2 cell differentiation were significantly enriched in AS. The significantly enriched KEGG pathways for DEGs are shown in Figure 1(a).

### 3.2. CD112R and CD96 Are Downregulated in AS Patients

Among the 9 picked IRs, CD112R and CD96 were significantly downregulated in AS patients (Figure 1(b)). CD112R and CD96 were also estimated by qPCR. The bioinformatics results revealed the same correlation between patients and control subjects. Also, the low expression of the two genes was observed in PBMCs from patients compared to healthy controls (Figure 1(c)).

### 3.3. Decreased Frequencies of Circulating CD96 Cells in AS Patients

**Figure 2:**
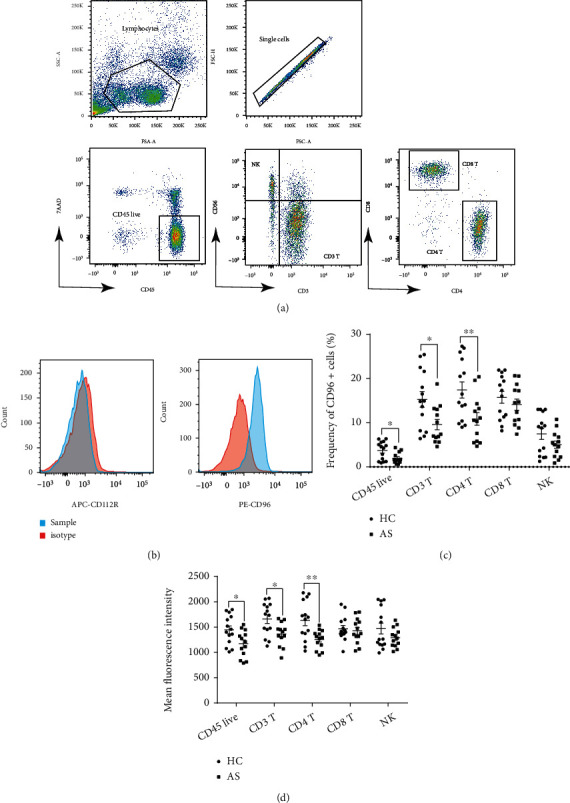
Flow cytometry. (a) Circling gate scheme of flow cytometry analysis. Gating lymphocytes according to the size and granularity (FSC and SSC). Circle the live immune cells “CD45 live” according to CD45 and 7AAD after obtaining single cells. In the gate of “CD45 live,” NK cells (CD3^−^CD56^+^) and conventional CD3 T cells (CD3^+^ CD56^−^) were sorted out. CD3 T cell (CD3^+^ CD56^−^) gate was divided into CD4 T and CD8 T cell subsets according to the expression of CD4 and CD8. (b) Histogram of the expression of CD112R and CD96 between samples and isotype. Compared to the isotype, the expression of CD112R increased slightly, but that of CD96 increased significantly. (c) Frequencies of each lymphocyte subset expressing CD96. The frequencies of CD96-positive CD45 live cells, CD3 T cells, and CD4 T cells in AS patients were lower than those in healthy controls, but no significant differences were observed on CD8 T and NK^−^ cells. (d) Comparison of the fluorescence intensities of CD96 expressed on lymphocyte subsets. The fluorescence intensities of CD96 expressed on CD45 live cells, CD3 T cells, and CD4^+^T cells were lower in AS patients than in healthy controls, but no significant differences were observed on CD8 T and NK cells (^∗^*P* < 0.05,  ^∗∗^*P* < 0.01).

We grouped the lymphocytes from PBMCs into several cell subgroups according to the cell surface markers (Figure 2(a)). CD96 was expressed normally on the CD45-positive live cells from fresh human blood, while CD112R was not expressed (Figure 2(b)). Compared to the healthy control samples, the frequencies of CD96-positive CD4 T cells in AS patients were significantly lower than in healthy controls (Figure 2(c)). The fluorescence intensities of CD96 expressed on peripheral blood lymphocytes of AS patients were significantly reduced compared to the controls. The differences were also observed in CD3 T and CD4 T cell subgroups (Figure 2(d)).

### 3.4. CD96 Knockdown Promotes the Inflammatory Response of CD4 T Cells

**Figure 3:**
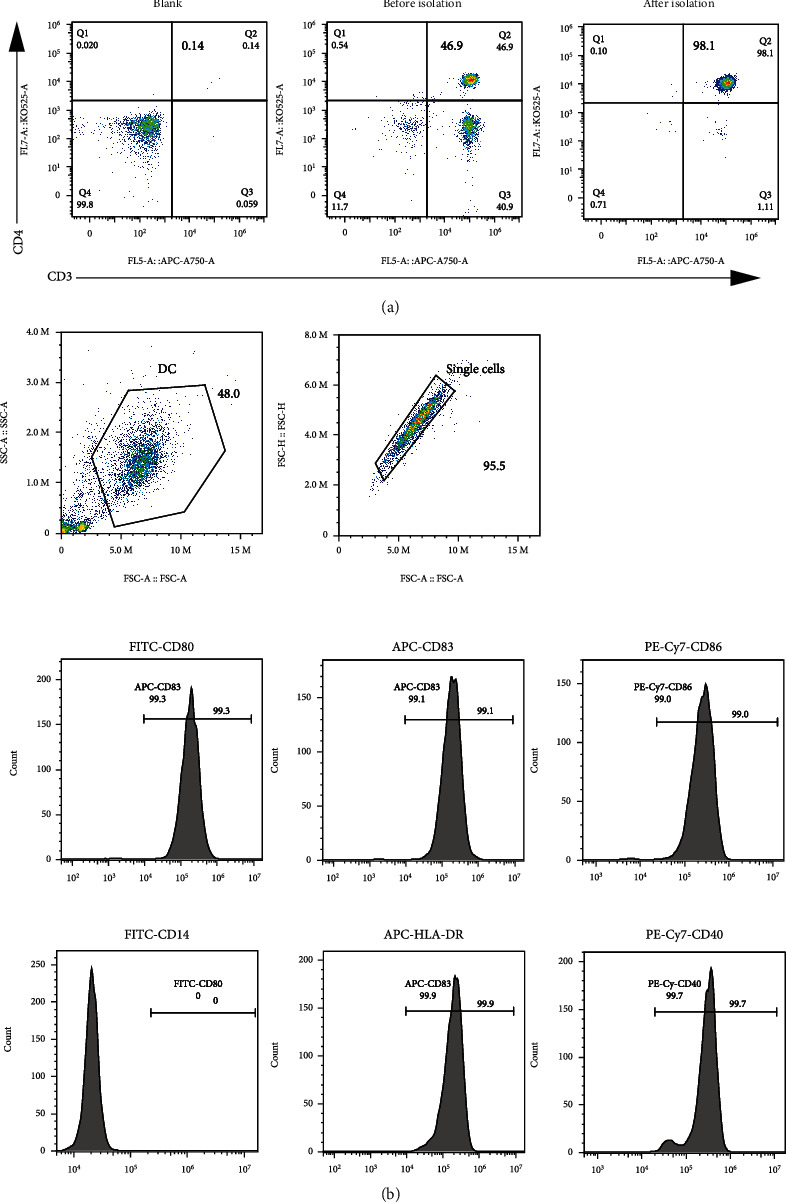
Purification and phenotype identification of CD4 T cells and mature DCs. (a) The purity of CD4 T cells was 46.9% before sorting and 98.1% after sorting. (b) Mature DCs expressed HLA-DR, CD40, CD80, CD83, and CD86 but did not express CD14.

**Figure 4:**
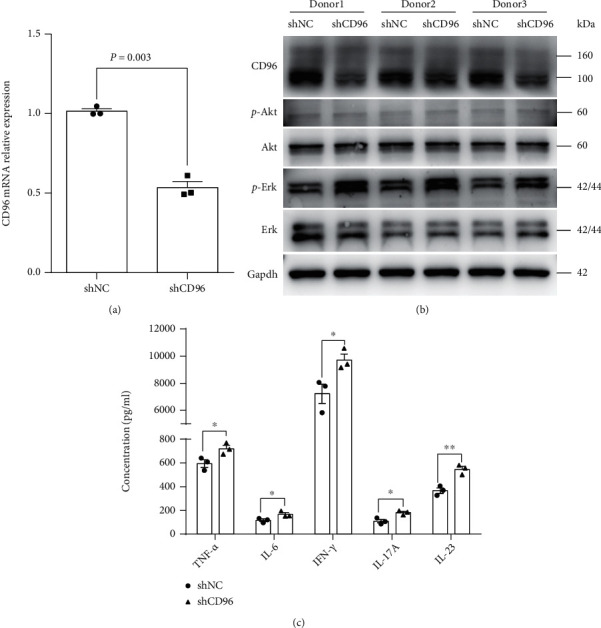
CD96 knockdown promoted the inflammatory response of CD4 T cells (three independent replicates were performed). (a) qPCR showed that the expression of *CD96* in the shCD96 group was significantly inhibited compared to shNC group. (b) Western blotting showed that the level of p-Erk/Erk was significantly increased in the shCD96 group compared to the shNC group, but there was no significant difference observed in p-Akt/Akt. (c) Compared to the shNC group, the levels of TNF-*α*, IL-6, IL-17A, IFN-*γ*, IL-23, and IFN-*γ* were significantly increased in the cell culture supernatants of the shCD96 group (^∗^*P* < 0.05,  ^∗∗^*P* < 0.01).

Before and after sorting human PBMCs with magnetic beads, the purity of CD4 T cells was 46.9% and 98.1% (Figure 3(a)), respectively. On day 7 post-DC stimulation, CD83, CD80, HLA-DR, CD86, and CD40, but not CD14, were highly expressed on the cell surface (Figure 3(b)). After CD4 T cells were transfected with the concentrated lentivirus for 72 h, qPCR and Western blotting showed that the expression of CD96 in the shCD96 group was significantly inhibited compared to the vector group (Figures 4(a) and 4(c)). CD4 T cells of shCD96 and shNC groups were cocultured with mature DCs for 5 days, respectively. Compared to the vector group, the levels of IL-17, TNF-*α*, IL-23, IL-6, and IFN-*γ* were significantly increased in the culture supernatant of the shCD96 group (Figure 4(b)), such that the level of cell proteins p-ERK/ERK, but not p-Akt/Akt, changed significantly (Figure 4(c)).

### 3.5. CD96 Overexpression Inhibits the Inflammatory Response of CD4 T Cells

**Figure 5:**
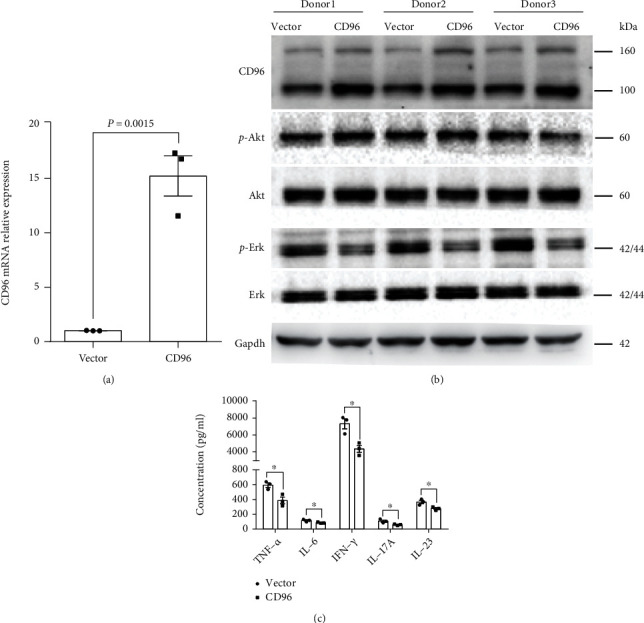
CD96 overexpression alleviated the inflammatory response of CD4 T cells (three independent replicates were performed). (a) qPCR showed that the expression of *CD96* in the CD96 group was significantly increased compared to the vector group. (b) Western blotting showed that the level of p-Erk/Erk was significantly downregulated in the CD96 group compared to vector group, but no significant difference was detected in p-Akt/Akt. (c) Compared to the vector group, the levels of TNF-*α*, IL-6, IFN-*γ*, IL-17A, and IL-23 were significantly decreased in the cell culture supernatants of the CD96 group (^∗^*P* < 0.05).

After CD4 T cells were transfected with concentrated lentivirus for 72 h, the expression of CD96 in the CD96 group was significantly overexpressed compared to the vector group, as assessed by qPCR (Figures 5(a) and 5(c)). CD4 T cells of CD96 and vector groups were cocultured with mature DCs for 5 days, respectively. Compared to those of the vector group, the levels of IL-17, TNF-*α*, IL-23, IL-6, interferon-gamma (IFN-*γ*), and p-ERK/ERK in the culture supernatant of the CD96 group were significantly decreased (Figure 5(b)). However, p-Akt/Akt showed no significant change (Figure 5(c)).

## 4. Discussion

In the present study, the T cell receptor (TCR) signaling pathway was significantly enriched in AS, and CD96 was significantly downregulated in CD4 T cells of AS patients. We also unveiled that low expression of CD96 promotes the immune response of CD4 T cells via the activated ERK signaling pathway. Consequently, CD96 downregulation might promote the pathogenesis of AS by boosting the immune response of CD4 T cells through the ERK signaling pathway.

TCR signaling is essential for the proliferation and function of T cells, while impaired TCR signaling may cause T cell-mediated autoimmune diseases [23]. Alterations in molecules that negatively regulate TCR signalling, such as Cbl- (Casitas B-lineage lymphoma-) family proteins, and alterations in the phos-phatidylinositol 3-kinase (PI3K) pathway, an T-cell signalling that is associated with survival, have been shown to play a role in promoting autoimmune disorders [24]. JNK pathway-associated phosphatase (JKAP), which can directly inactivates Lck by dephosphorylating tyrosine-394 residue during TCR signalling, was reported to be negatively correlates with clinical activity in intestinal bowel diseases (IBDs) [25] and systemic lupus erythematosus (SLE) [26]. JKAP-knockout mice show enhanced T-cell-mediated immune responses and are more susceptible to experimental autoimmune encephalomyelitis (EAE) [27]. In this study, the TCR signaling pathway was found to be significantly enriched in AS, indicating that components involved in this pathway might be associated with AS.

IRs are a class of negative regulators of TCR signaling pathway. A large number of studies have revealed the correlation between IRs and autoimmune diseases [28, 29]; CTLA4 and PD1 were associated with AS [9, 10]. Thus, it could be speculated that IRs are involved in the pathogenesis of AS. Bioinformatics analysis and qPCR showed decreased expression of both CD112R and CD96 in the PBMCs of AS patients. However, no abnormal expression of CTLA4 was detected but was highly expressed in AS patients [7, 8]. This result implied that the abnormal expression of CD112R and CD96 plays a role in the AS pathogenesis.

Additionally, the frequencies of CD96-positive CD4 T cells and the fluorescence intensities of CD96 expressed on CD4 T cells were lower in AS patients than in healthy controls. However, we did not detect significant CD112R expression on lymphocytes from fresh human blood by flow cytometry, while a significant population of T (CD3^+^) and NK (CD56^+^) cells has been shown to express low but detectable surface CD112R, as reported previously [30]. CD96 (TACTILE) [31] is a newly discovered member of the TIGIT receptor family, a class of immunoglobulin superfamily receptor clusters that interacts with ligands of the Nectin/Nectin-like family. These ligands, such as Nectin-2 (CD112) and Necl-5 (CD155 or PVR), are mainly expressed on different types of antigen presentation cells (APCs) [32]. The TIGIT receptor family includes DNAM-1, TIGIT, CD96, and CD112R, primarily expressed on the activated NK cells and T cells, and functions through specific motifs in the cytoplasmic domains [33]. Furthermore, DNAM-1 is a costimulatory receptor that activates the T and NK cells, with TIGIT, CD96, and CD112R as the coinhibitory receptors [34, 35].

CD96 was identified in 1992 [31] and found to be involved in some autoimmune diseases [36]. A genome-wide significant association between the CD96 locus and the production of antibodies to anti-TNF treatment was found in Crohn's disease (CD) [36]. A genome-wide association study revealed that CD96 was significantly associated with Mosaic chromosomal alterations (mCAs) in patients with CD [37]. Th9 cells and IL-9 are involved in the pathogenesis of IBDs [38]. CD96^high^ Th9 cells with increased IL-9 mRNA and protein expression showed less proliferation and migration potential, which could be restored by CD96 blockers [6].

However, the effect of CD96 on human lymphocytes is yet to be elucidated. Both human and mouse CD96 contains the ITIM-like domain, but human CD96 also contains a YXXM motif, frequently detected in activating coreceptors of the immunoglobulin superfamily, such as NKG2D and CD28; also, it might activate the PI3K/Akt pathway [39]. CD96 induces immunosuppression in mouse T and NK cells however, whether this receptor triggers inhibitory signal transduction or activates signal transduction in the human body remains unclear [40]. Chiang et al. [41] demonstrated that CD96 had a costimulatory effect on CD8 T cells. The activation signal of CD96 is transduced through MEK pathway, resulting in increased frequency of CD8 T cells expressing NUR77 and T-bet [41]. Conversely, recent studies have shown that CD96 has the opposite effect, especially in the regulation of T cell response. Blocking CD96 combined with other immune checkpoint inhibitors enhances T cell activity and inhibits tumor growth [42]. Blocking CD96-CD155 interaction increases NK cell cleavage of HepG2 cells [43]. Therefore, whether human CD96 activates or inhibits human T and NK cells needs to be clarified.

CD4 T cells play a major role in the pathogenesis of autoimmune diseases [44, 45], and the function of CD96 in CD4 T cells has not yet been reported. In this study, we knocked down the expression of CD96 on human CD4 T cells and cocultured them with OVA-pulsed mature DCs. The expression of TNF-*α*, IL-17A, IL-6, IL-23, and IFN-*γ* was significantly increased in the cell culture supernatants of the CD96 knockdown group. Western blot showed that the phosphorylation level of ERK was significantly elevated, but no significant difference was detected in p-Akt. This phenomenon suggested that low expression of CD96 promotes the immune response of CD4 T cells by activating the ERK signaling pathway. In addition, we overexpressed CD96 on human CD4 T cells and observed the opposite trend in proinflammatory cytokines and the phosphorylation level of ERK. The current findings are opposite to the results obtained by Chiang et al. on CD8 T cells [44], which could be attributed to different experimental conditions.

Based on these results, low expression of CD96 in peripheral blood CD4 T cells of AS patients and enhanced inflammatory response exhibited by CD96 knockdown CD4 T cells, we proposed two hypotheses: (1) decreased expression of CD96 weakens the inhibitory effect on T cell function, causing the progression of inflammation in AS; (2) inflammation causes CD96-positive cells to transfer to the diseased site or promote the consumption of CD96-positive cells, such that the proportion of CD96-positive cells is reduced. Battella et al. [46] demonstrated that the percentage of TIGIT-positive cells on intestinal mucosal T cells was significantly higher than that in the peripheral blood. This result indicated that the cells expressing TIGIT might accumulate to the lesion site under the influence of chemokines. Since the present study only observed CD96-positive cells in peripheral blood, the expression of CD96 in lesions of AS deserves further investigation.

## 5. Conclusions

In conclusion, our data indicated that the downregulation of CD96 on CD4 T is associated with AS. Also, CD96 might act as an inhibitory receptor on human CD4 T cells through ERK signaling pathway. The correlation between CD96 and AS needs to be studied further. Moreover, suitable animal models need to be developed to study the pathogenesis of autoimmune diseases caused by CD96 and provide evidence for the diagnosis, treatment, and prognosis of AS.

## Figures and Tables

**Table 1:** Clinical characteristics and laboratory features of patients with AS and healthy controls.

	Cohort I			Cohort II		
	AS (*n* = 19)	HC (*n* = 15)	*P*	AS (*n* = 13)	HC (*n* = 14)	*P*
Age (years)	41.8 ± 1.2 (20–52)	42.1 ± 0.9 (21–47)	0.4268	43.2 ± 0.9 (18–56)	42.8 ± 1.3 (20–53)	0.3789
Sex no. (%)	16 M/3 F	11 M/4 F	0.6722	10 M/3 F	11 M/3 F	0.918
Disease duration (years)	8.6 ± 0.5 (0.6 to 35)			9.0 ± 0.8 (0.5–37)		
Extra-articular manifestations (%)	5 (26)			4 (33.3)		
Peripheral arthritis (%)	6 (31.5)			5 (41.7)		
BASDAI (0–100)	34.6 ± 2.7 (0.4–94)			35.8 ± 2.4 (0.4–94)		
BASFI (0–100)	41.9 ± 3.0 (0–92)			40.2 ± 2.6 (0–92)		
HLA-B27^+^ (%)	15 (78.9)			10 (83.3)		
ESR (mm/h)	29.3 ± 2.6 (1–163)			28.0 ± 1.9 (1–163)		
CRP (mg/L)	25.3 ± 3.0 (0 to 350)			26.1 ± 3.2 (0–350)		

Data are expressed as the mean ± standard error of the mean (range). The Mann–Whitney *U* test was used to compare the age, and the chi-square test was used to compare sex between the two groups. BASDAI: Bath Ankylosing Spondylitis Disease Activity Index; BASFI: Bath Ankylosing Spondylitis Functional Index; CRP: C-reactive protein; ESR: erythrocyte sedimentation rate.

## Data Availability

The datasets used and analyzed during the current study are available from the corresponding author upon reasonable request.

## Supplementary Materials

Supplementary Materials Table S1: 642 DEGs in AS patients.

## References

[1] Wordsworth B. P., Cohen C. J., Davidson C., Vecellio M. (2021). Perspectives on the genetic associations of ankylosing spondylitis. *Frontiers in Immunology*.

[2] Cortes A., Hadler J., Pointon J. P. (2013). Identification of multiple risk variants for ankylosing spondylitis through high-density genotyping of immune-related loci. *Nature genetics*.

[3] Evans D. M., Spencer C. C., Pointon J. J. (2011). Interaction between ERAP1 and HLA-B27 in ankylosing spondylitis implicates peptide handling in the mechanism for HLA-B27 in disease susceptibility. *Nature Genetics*.

[4] Grebinoski S., Vignali D. A. (2020). Inhibitory receptor agonists: the future of autoimmune disease therapeutics?. *Current Opinion in Immunology*.

[5] Anderson A. C., Joller N., Kuchroo V. K. (2016). Lag-3, Tim-3, and TIGIT: co-inhibitory receptors with specialized functions in immune regulation. *Immunity*.

[6] Stanko K., Iwert C., Appelt C. (2018). CD96 expression determines the inflammatory potential of IL-9–producing Th9 cells. *Proceedings of the National Academy of Sciences*.

[7] Çetintepe S. P., Şentürk T., Sargın G., Aydın N. (2018). Serum sCTLA-4 levels and clinical manifestations in ankylosing spondylitis patients. *European Journal of Rheumatology*.

[8] Toussirot E., Saas P., Deschamps M. (2009). Increased production of soluble CTLA-4 in patients with spondylarthropathies correlates with disease activity. *Arthritis Research & Therapy*.

[9] Romo-Tena J., Gómez-Martín D., Alcocer-Varela J. (2013). CTLA-4 and autoimmunity: new insights into the dual regulator of tolerance. *Autoimmunity Reviews*.

[10] Zamani M. R., Aslani S., Salmaninejad A., Javan M. R., Rezaei N. (2016). PD-1/PD-L and autoimmunity: a growing relationship. *Cellular Immunology*.

[11] Abrams J. R., Lebwohl M. G., Guzzo C. A. (1999). CTLA4Ig-mediated blockade of T-cell costimulation in patients with psoriasis vulgaris. *The Journal of Clinical Investigation*.

[12] Kremer J. M., Westhovens R., Leon M. (2003). Treatment of rheumatoid arthritis by selective inhibition of T-cell activation with fusion protein CTLA4Ig. *New England Journal of Medicine*.

[13] Song I. H., Heldmann F., Rudwaleit M. (2011). Treatment of active ankylosing spondylitis with abatacept: an open-label, 24-week pilot study. *Annals of the Rheumatic Diseases*.

[14] Eric Gracey Y. Y. (2016). sexual dimorphism in the Th17 signature of ankylosing spondylitis. *Arthritis & Rhematology*.

[15] Pimentel-Santos F. M., Ligeiro D., Matos M. (2011). Whole blood transcriptional profiling in ankylosing spondylitis identifies novel candidate genes that might contribute to the inflammatory and tissue-destructive disease aspects. *Arthritis Research & Therapy*.

[16] Buckle I., Guillerey C. (2021). Inhibitory receptors and immune checkpoints regulating natural killer cell responses to cancer. *Cancers*.

[17] Livak K. J., Schmittgen T. D. (2001). Analysis of relative gene expression data using real-time quantitative PCR and the 2− *ΔΔ*CT method. *methods*.

[18] Valhondo I., Hassouneh F., Lopez-Sejas N. (2020). Characterization of the DNAM-1, TIGIT and TACTILE axis on circulating NK, NKT-like and T cell subsets in patients with acute myeloid leukemia. *Cancers*.

[19] Williams C. A., Harry R. A., McLeod J. D. (2008). Apoptotic cells induce dendritic cell-mediated suppression via interferon-gamma-induced IDO. *Immunology*.

[20] Zhang X., Huang H., Yuan J. (2005). CD4−8− dendritic cells prime CD4+ T regulatory 1 cells to suppress antitumor immunity. *The Journal of Immunology*.

[21] Yokosuka T., Takamatsu M., Kobayashi-Imanishi W., Hashimoto-Tane A., Azuma M., Saito T. (2012). Programmed cell death 1 forms negative costimulatory microclusters that directly inhibit T cell receptor signaling by recruiting phosphatase SHP2. *The Journal of Experimental Medicine*.

[22] Blom R. A. M., Amacker M., Moser C. (2017). Virosome-bound antigen enhances DC-dependent specific CD4(+) T cell stimulation, inducing a Th1 and Treg profile in vitro. *Nanomedicine*.

[23] Takeuchi Y., Hirota K., Sakaguchi S. (2020). Impaired T cell receptor signaling and development of T cell–mediated autoimmune arthritis. *Immunological Reviews*.

[24] Ohashi P. S. (2002). T-cell signalling and autoimmunity: molecular mechanisms of disease. *Nature Reviews. Immunology*.

[25] Zhou R., Chang Y., Liu J. (2017). JNK pathway-associated phosphatase/DUSP22 suppresses CD4(+) T-cell activation and Th1/Th17-cell differentiation and negatively correlates with clinical activity in inflammatory bowel disease. *Frontiers in Immunology*.

[26] Chuang H.-C., Tan T.-H. (2019). MAP4K family kinases and DUSP family phosphatases in T-cell signaling and systemic lupus erythematosus. *Cell*.

[27] Li J. P., Yang C. Y., Chuang H. C. (2014). The phosphatase JKAP/DUSP22 inhibits T-cell receptor signalling and autoimmunity by inactivating Lck. *Nature Communications*.

[28] Lavon I., Heli C., Brill L., Charbit H., Vaknin-Dembinsky A. (2019). Blood levels of co-inhibitory-receptors: a biomarker of disease prognosis in multiple sclerosis. *Frontiers in Immunology*.

[29] Schnell A., Bod L., Madi A., Kuchroo V. K. (2020). The yin and yang of co-inhibitory receptors: toward anti-tumor immunity without autoimmunity. *Cell Research*.

[30] Zhu Y., Paniccia A., Schulick A. C. (2016). Identification of CD112R as a novel checkpoint for human T cells. *The Journal of Experimental Medicine*.

[31] Wang P. L., O'Farrell S., Clayberger C., Krensky A. M. (1992). Identification and molecular cloning of tactile. A novel human T cell activation antigen that is a member of the Ig gene superfamily. *Journal of Immunology*.

[32] Stamm H., Wellbrock J., Fiedler W. (2018). Interaction of PVR/PVRL2 with TIGIT/DNAM-1 as a novel immune checkpoint axis and therapeutic target in cancer. *Mammalian Genome*.

[33] Lozano E., Dominguez-Villar M., Kuchroo V., Hafler D. A. (2012). The TIGIT/CD226 axis regulates human T cell function. *Journal of Immunology*.

[34] Jin H. S., Park Y. (2021). Hitting the complexity of the TIGIT-CD96-CD112R-CD226 axis for next-generation cancer immunotherapy. *BMB Reports*.

[35] Sanchez-Correa B., Valhondo I., Hassouneh F. (2019). DNAM-1 and the TIGIT/PVRIG/TACTILE axis: novel immune checkpoints for natural killer cell-based cancer immunotherapy. *Cancers (Basel)*.

[36] Aterido A., Palau N., Domènech E. (2019). Genetic association between CD96 locus and immunogenicity to anti-TNF therapy in Crohn’s disease. *The pharmacogenomics journal*.

[37] Kakuta Y., Iwaki H., Umeno J. (2022). Crohn’s disease and early exposure to thiopurines are independent risk factors for mosaic chromosomal alterations in patients with inflammatory bowel diseases. *Journal of Crohn's and Colitis*.

[38] Nalleweg N., Chiriac M. T., Podstawa E. (2015). IL-9 and its receptor are predominantly involved in the pathogenesis of UC. *Gut*.

[39] Chambers C. A. (2001). The expanding world of co-stimulation: the two-signal model revisited. *Trends in Immunology*.

[40] Georgiev H., Ravens I., Papadogianni G., Bernhardt G. (2018). Coming of age: CD96 emerges as modulator of immune responses. *Frontiers in Immunology*.

[41] Chiang E. Y., de Almeida P. E., de Almeida Nagata D. E. (2020). CD96 functions as a co-stimulatory receptor to enhance CD8+T cell activation and effector responses. *European Journal of Immunology*.

[42] Roman Aguilera A., Lutzky V. P., Mittal D. (2018). CD96 targeted antibodies need not block CD96-CD155 interactions to promote NK cell anti-metastatic activity. *Oncoimmunology*.

[43] Sun H., Huang Q., Huang M. (2019). Human CD96 correlates to natural killer cell exhaustion and predicts the prognosis of human hepatocellular carcinoma. *Hepatology*.

[44] Boyle L. H., Hill Gaston J. S. (2003). Breaking the rules: the unconventional recognition of HLA‐B27 by CD4+ T lymphocytes as an insight into the pathogenesis of the spondyloarthropathies. *Rheumatology*.

[45] Jansen D. T., Hameetman M., van Bergen J. (2015). IL-17-producing CD4+ T cells are increased in early, active axial spondyloarthritis including patients without imaging abnormalities. *Rheumatology*.

[46] Battella S., Oliva S., Franchitti L. (2019). Fine tuning of the DNAM-1/TIGIT/ligand axis in mucosal T cells and its dysregulation in pediatric inflammatory bowel diseases (IBD). *Mucosal Immunology*.

