# Supplementary material for: CD96 Downregulation Promotes the Immune Response of CD4 T Cells and Associates with Ankylosing Spondylitis
Source: Biomed Res Int. 2022 Jun 19;2022:3946754. doi: 10.1155/2022/3946754 (PMC9234051; doi:10.1155/2022/3946754)
Supplement: Supplementary Materials — Table S1: 642 DEGs in AS patients. [file 3946754.f1.pdf]

gene	logFC	AveExpr	t	P.Value	adj.P.Val	B
ACTG1	-0.322623	13.101459	-6.960438	3.03E-10	1.57E-06	12.909921
IL2RB	-0.5855	10.187548	-6.316045	6.58E-09	1.71E-05	10.04788
CD81	-0.397247	11.780627	-6.228428	9.90E-09	1.71E-05	9.6672773
TAB2	0.2054918	7.4402644	6.0988542	1.81E-08	2.34E-05	9.1086468
RBM47	0.3984527	8.6391003	5.9155894	4.19E-08	4.34E-05	8.3276276
RPL3	-0.331369	10.93164	-5.644732	1.42E-07	0.0001226	7.1943277
GZMM	-0.385523	8.071595	-5.559814	2.07E-07	0.000147	6.8445348
ZDHHC18	0.4112692	9.9846702	5.5381097	2.28E-07	0.000147	6.7555721
JMJD8	-0.256721	10.045747	-5.512343	2.55E-07	0.000147	6.6501927
PKN2	0.2705285	8.4208108	5.4834333	2.90E-07	0.0001502	6.5322723
CD247	-0.400824	11.7512	-5.335363	5.53E-07	0.0002548	5.9335312
EXOSC10	-0.240297	9.0780313	-5.320473	5.90E-07	0.0002548	5.8738173
TP53INP1	0.3109598	9.5586046	5.2397672	8.35E-07	0.0003205	5.5517771
ASMTL	-0.189178	8.1138495	-5.231431	8.65E-07	0.0003205	5.518669
STAT5B	0.2546816	9.2230485	5.1335446	1.31E-06	0.0004197	5.1321714
LCOR	0.1956147	8.4932744	5.1282961	1.34E-06	0.0004197	5.1115665
EI24	-0.170268	8.126197	-5.122699	1.38E-06	0.0004197	5.0896075
BLOC1S4	-0.163366	8.3164071	-5.036998	1.98E-06	0.0005673	4.7551112
RASSF5	-0.14978	9.5335478	-5.011141	2.20E-06	0.0005673	4.6548415
OSBP	-0.174493	9.2452166	-5.01095	2.20E-06	0.0005673	4.6541028
ADRB2	-0.29953	8.9202159	-4.995507	2.35E-06	0.0005673	4.5943658
SLU7	0.2482754	8.6119023	4.9899528	2.41E-06	0.0005673	4.5729073
EWSR1	-0.162404	9.1464978	-4.94348	2.92E-06	0.0006587	4.3939243
FAM43A	-0.207352	8.045673	-4.884478	3.73E-06	0.0008062	4.1681473
SLC9A3R1	-0.266561	10.14247	-4.867141	4.01E-06	0.0008205	4.1021193
SLA	0.2772993	10.990686	4.860831	4.11E-06	0.0008205	4.078123
PDZD8	0.1991568	7.5760891	4.8134265	5.00E-06	0.0009532	3.8984654
TINF2	-0.227354	10.095405	-4.806313	5.15E-06	0.0009532	3.8715997
CMTM7	-0.202877	11.24995	-4.788475	5.54E-06	0.0009723	3.8043409
PLEKHF1	-0.324427	8.643154	-4.78459	5.62E-06	0.0009723	3.7897124
AKR1B1	-0.241254	9.5941643	-4.757287	6.29E-06	0.0010517	3.687119
QRICH1	-0.1883	9.2363382	-4.727806	7.09E-06	0.0011486	3.5767571
LRRC41	-0.167786	7.5156317	-4.719255	7.34E-06	0.0011531	3.5448266
DUSP14	-0.132285	7.208672	-4.705955	7.74E-06	0.0011811	3.4952362
C16orf58	-0.235931	8.9547605	-4.69064	8.24E-06	0.0012206	3.4382391
SLC3A2	-0.149287	7.7255347	-4.663171	9.20E-06	0.0013257	3.3363118
ZNF281	0.261402	9.5534243	4.6406288	1.01E-05	0.0014121	3.2529535
FEM1C	0.2341908	8.4602148	4.6211475	1.09E-05	0.0014361	3.1811222
PSMC1	-0.146474	10.490063	-4.617474	1.11E-05	0.0014361	3.1675991
LBH	-0.247879	8.9288239	-4.61697	1.11E-05	0.0014361	3.1657435
SUMO2	-0.269085	9.8446469	-4.600524	1.18E-05	0.0014963	3.1052916
ATP6V1C1	0.175993	7.9155848	4.5931433	1.22E-05	0.0015043	3.0782072
CLK4	0.2260183	7.8431303	4.5757299	1.31E-05	0.0015653	3.0144175
ALKBH5	-0.176181	11.282125	-4.571485	1.33E-05	0.0015653	2.9988908
MED13L	0.1790051	7.3542882	4.5577267	1.40E-05	0.0016069	2.9486335
DEXI	-0.184205	8.7094774	-4.550787	1.44E-05	0.0016069	2.9233201
CDC37	-0.212836	11.624295	-4.54173	1.49E-05	0.0016069	2.8903226
AIMP2	-0.153937	8.6302341	-4.54164	1.50E-05	0.0016069	2.8899958
SKA2	0.1037428	6.9310823	4.537774	1.52E-05	0.0016069	2.8759238
SQSTM1	-0.232449	10.825283	-4.530221	1.56E-05	0.0016225	2.8484559

EID2	-0.155511	7.2513897	-4.510417	1.69E-05	0.0017199	2.7765732
ZNF329	-0.13648	7.7668634	-4.505537	1.72E-05	0.0017199	2.7588931
TMEM35B	-0.201094	9.4350318	-4.500499	1.76E-05	0.0017213	2.7406502
MAPK7	-0.15033	7.5050624	-4.489291	1.84E-05	0.0017657	2.7001223
SBDSP1	-0.082654	6.9353111	-4.4644	2.03E-05	0.0018808	2.6103511
SNU13	-0.173349	8.9192283	-4.455644	2.10E-05	0.0018808	2.5788489
CPSF4	-0.155606	9.3113702	-4.455478	2.10E-05	0.0018808	2.5782543
DCP2	0.2684234	10.103867	4.455006	2.10E-05	0.0018808	2.576557
SBK1	-0.229326	8.175858	-4.445955	2.18E-05	0.0018898	2.5440433
GALT	-0.157146	7.9358975	-4.445124	2.19E-05	0.0018898	2.5410593
ZNF207	-0.152202	9.1217316	-4.440538	2.23E-05	0.0018925	2.5246035
LRRC47	-0.140406	8.297642	-4.413186	2.48E-05	0.0020717	2.4266926
CS	-0.182628	10.190726	-4.406274	2.54E-05	0.0020944	2.402014
ADGRG5	-0.249649	8.1090813	-4.394517	2.66E-05	0.0021581	2.3600985
ALG8	-0.130158	8.1803509	-4.384076	2.77E-05	0.0022128	2.3229356
KCTD5	-0.152738	8.8450853	-4.37881	2.83E-05	0.0022242	2.3042164
ATIC	-0.23975	9.636209	-4.360629	3.04E-05	0.0023337	2.2396996
TMEM41A	-0.097972	7.2356448	-4.358687	3.06E-05	0.0023337	2.2328196
NOP56	-0.221653	9.1462804	-4.348092	3.19E-05	0.0023711	2.1953174
VCPIP1	0.250706	8.329586	4.3470651	3.20E-05	0.0023711	2.1916872
HCST	-0.254092	12.504618	-4.327237	3.45E-05	0.0025234	2.1216835
PICALM	0.2724997	10.896712	4.3083596	3.71E-05	0.0026591	2.0552406
COP1	0.198008	10.503046	4.306376	3.74E-05	0.0026591	2.0482703
DCTN2	-0.122285	10.166697	-4.257057	4.52E-05	0.0031676	1.8756784
TBC1D19	-0.074432	6.8619913	-4.247593	4.69E-05	0.0032401	1.8427158
PAFAH2	-0.160987	8.1171317	-4.238265	4.85E-05	0.0033011	1.8102747
AARS	-0.259904	9.3713102	-4.235759	4.90E-05	0.0033011	1.8015689
KLRG1	-0.438985	9.7645556	-4.219433	5.21E-05	0.0034277	1.7449342
ACSL1	0.440342	10.755203	4.218673	5.23E-05	0.0034277	1.7423029
NT5C2	0.207704	10.245414	4.2157603	5.29E-05	0.0034277	1.7322163
PRPF4	-0.11557	8.6132743	-4.206397	5.48E-05	0.0035075	1.6998233
HLA-DRA	-0.240847	13.143962	-4.202249	5.56E-05	0.0035195	1.6854918
HBP1	0.1665334	9.4097287	4.1837884	5.97E-05	0.003728	1.6218183
LUZP1	-0.150471	7.8786803	-4.17758	6.11E-05	0.0037344	1.6004474
NONO	-0.133236	9.0626908	-4.175486	6.16E-05	0.0037344	1.5932464
PPP2R2B	-0.306417	8.2359636	-4.171211	6.26E-05	0.0037344	1.5785503
RXYLT1	-0.13513	8.1777172	-4.165988	6.38E-05	0.0037344	1.5606087
CCT3	-0.178134	8.783743	-4.163035	6.45E-05	0.0037344	1.5504712
HDHD5	-0.158261	8.6747969	-4.162804	6.46E-05	0.0037344	1.5496809
PROS1	0.3501196	7.5756388	4.1561716	6.62E-05	0.0037344	1.5269321
RHOC	-0.243134	9.3385901	-4.15601	6.62E-05	0.0037344	1.5263773
ACSL4	0.3106725	8.9319857	4.1559237	6.63E-05	0.0037344	1.5260825
WDR1	-0.184582	10.079469	-4.147166	6.85E-05	0.0037344	1.4960868
TMEM138	-0.11909	7.8823457	-4.146428	6.87E-05	0.0037344	1.4935598
MAP4K1	-0.267524	9.7595347	-4.145479	6.89E-05	0.0037344	1.490314
PYGL	0.3610466	10.514456	4.1411704	7.00E-05	0.0037344	1.4755764
KCNJ15	0.4018793	8.6191032	4.1385775	7.07E-05	0.0037344	1.4667131
PHF21A	0.2280901	10.56462	4.1341353	7.19E-05	0.0037344	1.4515371
PVRIG	-0.267339	9.2377093	-4.13405	7.19E-05	0.0037344	1.4512472
NELFCD	-0.17356	8.9447999	-4.131705	7.26E-05	0.0037344	1.4432384
HNRNPK	-0.277729	9.8093592	-4.131053	7.27E-05	0.0037344	1.4410147

E2F3	0.2556374	9.141965	4.1174833	7.65E-05	0.0038901	1.3947518
HNMT	0.2024908	7.3788637	4.1136582	7.76E-05	0.0039077	1.3817308
SPOCK2	-0.317509	11.404793	-4.108655	7.91E-05	0.0039405	1.3647122
UTP14A	-0.115971	7.9220482	-4.106256	7.98E-05	0.0039405	1.3565571
FBLN5	-0.192031	7.4474362	-4.09114	8.44E-05	0.0041292	1.3052503
SEC22C	-0.127433	7.8448401	-4.087657	8.55E-05	0.0041439	1.2934481
SNAPC2	-0.107393	7.8527601	-4.081346	8.75E-05	0.0041718	1.27208
IFRD1	0.2945627	8.5150672	4.0808575	8.77E-05	0.0041718	1.2704258
PTGER2	-0.220945	8.6221978	-4.066449	9.25E-05	0.0043607	1.2217348
YPEL5	0.2554635	10.404301	4.0569531	9.58E-05	0.0044238	1.1897101
NSMF	-0.180147	7.6439499	-4.056696	9.59E-05	0.0044238	1.1888441
ARHGEF18	-0.201502	11.200205	-4.055006	9.65E-05	0.0044238	1.1831504
CNOT11	-0.158465	9.7342137	-4.051285	9.78E-05	0.0044238	1.1706194
USP15	0.2529457	8.331074	4.0505431	9.81E-05	0.0044238	1.1681237
GNAI3	0.219341	8.4861002	4.0466002	9.95E-05	0.004427	1.1548577
RNPS1	-0.146363	9.1991586	-4.045354	1.00E-04	0.004427	1.1506654
TAPT1	0.1348803	7.5599531	4.0433667	0.0001007	0.004427	1.1439853
SNIP1	-0.126046	7.7140993	-4.032291	0.0001049	0.0045727	1.1067918
KIAA2026	-0.121525	7.4148429	-4.025131	0.0001077	0.0046245	1.082788
GZMK	-0.462137	9.8474629	-4.021881	0.000109	0.0046245	1.0719011
SH2D1B	-0.319564	7.8484536	-4.020968	0.0001094	0.0046245	1.0688438
UGP2	0.1596133	8.7534878	4.0177133	0.0001107	0.0046245	1.05795
SAMM50	-0.201465	9.0324027	-4.017108	0.000111	0.0046245	1.0559248
RALGDS	-0.267502	10.081132	-4.015859	0.0001115	0.0046245	1.0517459
CXCR6	-0.175032	7.1661239	-4.010628	0.0001136	0.0046767	1.0342557
AK5	-0.098344	6.8475424	-4.004439	0.0001162	0.0047464	1.0135865
GNLY	-0.589745	11.533635	-3.982245	0.0001261	0.0050921	0.9396481
OGFOD1	-0.121388	8.2624439	-3.980958	0.0001267	0.0050921	0.9353698
TOMM40	-0.172521	8.8670588	-3.958051	0.0001377	0.0054931	0.8593883
CCDC89	0.0983903	7.1695088	3.9537092	0.0001399	0.0055122	0.8450225
ABLIM3	0.1795795	7.146592	3.9528991	0.0001403	0.0055122	0.8423433
DNAJC8	-0.182823	10.50084	-3.939568	0.0001473	0.0057195	0.7983129
TGS1	-0.099907	6.9646848	-3.936982	0.0001487	0.0057195	0.7897837
NUDT9	-0.12414	7.6493973	-3.936543	0.0001489	0.0057195	0.7883384
NUMB	0.251175	10.494969	3.9316381	0.0001516	0.0057793	0.7721736
HOMER2	0.1583224	7.1110281	3.9269054	0.0001542	0.0058364	0.7565914
ZBTB34	0.2216133	8.7106658	3.9210974	0.0001575	0.0059171	0.7374879
CYB5R4	0.2653391	9.2645826	3.9101916	0.0001638	0.0061107	0.7016722
JAK2	0.2132132	7.7514637	3.9016431	0.0001689	0.0062289	0.6736495
GPR89B	-0.134924	7.2887604	-3.899618	0.0001702	0.0062289	0.6670176
ACTR1B	-0.101317	8.3776747	-3.898961	0.0001706	0.0062289	0.6648653
DNAJA3	-0.15431	8.9563926	-3.893378	0.000174	0.006311	0.6465976
MIS18A	-0.094652	6.9868366	-3.889002	0.0001768	0.0063667	0.6322942
EXOC6	0.1930712	8.0325195	3.8775504	0.0001842	0.0065885	0.5949141
CFL1	-0.151612	11.998599	-3.871344	0.0001884	0.0066628	0.57469
CENPB	-0.194345	9.5922265	-3.87061	0.0001889	0.0066628	0.572301
ZNF263	-0.111515	8.1527764	-3.865696	0.0001922	0.0067354	0.5563069
SYNJ1	0.1517531	8.1390591	3.8574465	0.000198	0.0068908	0.5294897
KCMF1	0.1065688	7.0706445	3.8517286	0.0002021	0.0069462	0.5109275
AKR7A2	-0.155311	9.5860104	-3.851481	0.0002023	0.0069462	0.510126
PLEKHA1	-0.27343	9.5789938	-3.846568	0.0002058	0.0070228	0.4941928

PTPN1	-0.174711	9.5478852	-3.840293	0.0002105	0.0071256	0.473866
CD96	-0.25082	9.3412597	-3.838836	0.0002116	0.0071256	0.4691492
TNNC2	0.1581036	7.0337833	3.8363824	0.0002135	0.0071333	0.4612105
LPIN1	-0.215309	9.1168189	-3.834915	0.0002146	0.0071333	0.4564633
RPL7L1	-0.156515	10.79284	-3.827342	0.0002204	0.0072817	0.4319913
CHD7	0.1476366	7.9975286	3.8038934	0.0002396	0.0078644	0.3564428
NKG7	-0.467017	11.897227	-3.797812	0.0002448	0.0079375	0.3369049
CDK4	-0.134477	8.6805446	-3.796235	0.0002462	0.0079375	0.3318443
MIPEP	-0.080188	7.0411131	-3.795978	0.0002464	0.0079375	0.3310178
FKBP1A	0.2540053	10.51599	3.7929256	0.0002491	0.0079519	0.3212248
NUCKS1	-0.185988	8.5567296	-3.790368	0.0002514	0.0079519	0.3130229
GALK2	-0.108296	7.8225343	-3.790249	0.0002515	0.0079519	0.3126417
TMEM69	-0.104784	8.0504224	-3.784993	0.0002562	0.008052	0.2958004
REXO4	-0.158758	7.7887342	-3.776728	0.0002638	0.0082329	0.2693527
PSMB10	-0.164839	12.163025	-3.775292	0.0002651	0.0082329	0.2647612
CIR1	0.1662914	8.7151289	3.7732514	0.000267	0.0082431	0.2582406
RUNX2	0.1375834	7.4656461	3.7677941	0.0002722	0.0083535	0.2408135
NUBP1	-0.100128	7.8522208	-3.759886	0.0002799	0.0084975	0.215595
DOLPP1	-0.084972	7.2706176	-3.759602	0.0002802	0.0084975	0.2146902
PSMA1	-0.139258	10.121717	-3.756517	0.0002833	0.0085403	0.20486
MRRF	-0.091136	7.7797166	-3.750956	0.0002888	0.0086507	0.1871616
EIF3G	-0.196913	10.818945	-3.74918	0.0002907	0.0086507	0.1815138
NFAT5	-0.218133	7.5746281	-3.747945	0.0002919	0.0086507	0.1775872
ACSL5	-0.154632	9.1222348	-3.746321	0.0002936	0.0086507	0.1724272
PUS1	-0.18229	8.8324146	-3.737861	0.0003024	0.008861	0.145562
GSTP1	-0.171596	10.936238	-3.734789	0.0003057	0.0089066	0.1358203
UBIAD1	-0.12263	7.7197585	-3.728317	0.0003127	0.0090599	0.1153125
GPCPD1	0.2215788	8.0957385	3.7204415	0.0003214	0.0092612	0.0903963
RBM14	-0.153513	9.4333724	-3.704336	0.00034	0.0097426	0.0395632
CBLB	-0.209946	8.7596448	-3.701323	0.0003436	0.0097914	0.0300715
CUTA	-0.222105	10.409454	-3.695169	0.0003511	0.0099488	0.0107051
LINGO2	-0.104924	6.7871104	-3.692124	0.0003548	0.01	0.0011335
NSUN4	-0.100191	7.8552361	-3.684091	0.0003648	0.0102276	-0.024097
EAF2	0.1978169	7.2907177	3.6741383	0.0003777	0.0104213	-0.055295
RPL10	-0.165347	14.495174	-3.673514	0.0003785	0.0104213	-0.05725
SCAF4	-0.162389	7.888604	-3.673402	0.0003786	0.0104213	-0.057601
SAE1	-0.167555	10.320141	-3.672516	0.0003798	0.0104213	-0.060373
GMFG	0.2034146	11.958098	3.6668851	0.0003873	0.0105707	-0.077991
LPGAT1	0.16962	7.7427607	3.6629093	0.0003926	0.0106053	-0.090417
ZNF615	-0.095142	7.6301987	-3.662464	0.0003933	0.0106053	-0.09181
TREML1	0.2660799	7.5993197	3.6614124	0.0003947	0.0106053	-0.095093
SVIL	0.2328273	9.7463901	3.656389	0.0004016	0.0107355	-0.110774
SCYL2	0.1445678	8.4631737	3.6436539	0.0004196	0.0111212	-0.150454
TUBB	-0.15739	10.023047	-3.64319	0.0004203	0.0111212	-0.151897
ELP4	-0.075906	7.1833888	-3.640234	0.0004246	0.011178	-0.161092
ADCK2	-0.128604	8.1231081	-3.636409	0.0004303	0.0112691	-0.172982
C6orf62	0.1987094	10.536988	3.6311225	0.0004381	0.0114183	-0.189396
EIF4A3	-0.145948	9.96457	-3.628966	0.0004414	0.0114457	-0.196086
AIFM1	-0.104675	7.7258733	-3.62734	0.0004439	0.0114527	-0.201131
TIGD5	-0.115851	7.5535397	-3.621972	0.0004521	0.0116081	-0.217765
CAVIN2	0.4097761	9.8697375	3.6159837	0.0004615	0.0117907	-0.236303

ODF2	-0.09319	7.5643336	-3.614138	0.0004645	0.0118075	-0.242012
CA5B	-0.18086	7.8912906	-3.606527	0.0004767	0.0120603	-0.265529
DOK3	0.1499199	7.4544724	3.6023846	0.0004835	0.0121629	-0.278311
ERGIC2	0.1819319	7.7664181	3.6012157	0.0004855	0.0121629	-0.281916
RPL18	-0.269195	12.549664	-3.594532	0.0004967	0.0123842	-0.302513
PXN	0.1827086	8.1376185	3.5917674	0.0005014	0.0123905	-0.311023
GOT1	-0.122901	7.9593085	-3.591581	0.0005017	0.0123905	-0.311598
RNF149	0.2509733	12.383442	3.5831377	0.0005164	0.0126422	-0.337557
ALCAM	0.1672868	7.7130316	3.5829081	0.0005168	0.0126422	-0.338262
SEC31A	-0.165596	8.1152891	-3.576845	0.0005276	0.0128455	-0.356873
GPBP1	0.1653047	9.3692701	3.5705829	0.000539	0.0130576	-0.376071
TM9SF3	0.1364652	8.2071187	3.5692866	0.0005413	0.0130576	-0.380042
LAMA3	0.0720852	6.9370962	3.5628837	0.0005532	0.0132345	-0.399638
IRF8	-0.228746	9.8365985	-3.56236	0.0005542	0.0132345	-0.401241
ZNF428	-0.17975	9.9752387	-3.560972	0.0005569	0.0132345	-0.405484
VAMP3	0.1451774	9.933089	3.5593014	0.00056	0.0132345	-0.41059
HTATIP2	0.1795472	9.3009587	3.5585573	0.0005614	0.0132345	-0.412864
ZSCAN18	-0.212329	8.7045435	-3.554799	0.0005686	0.013251	-0.424343
HLA-DMA	-0.241746	10.844563	-3.553486	0.0005712	0.013251	-0.428351
SMG7	0.1691827	9.094336	3.5528306	0.0005724	0.013251	-0.430351
PTGDS	-0.401857	7.9659442	-3.551811	0.0005744	0.013251	-0.433461
TLR4	0.2686198	8.5846637	3.5515644	0.0005749	0.013251	-0.434215
IK	-0.133562	9.3727757	-3.546796	0.0005843	0.0133609	-0.448755
SEC61A1	-0.170243	10.0282	-3.54632	0.0005852	0.0133609	-0.450206
ANXA7	-0.135696	9.7040873	-3.544113	0.0005896	0.0133609	-0.456929
STK25	-0.109488	8.2672665	-3.54262	0.0005926	0.0133609	-0.461476
LGR6	-0.13299	7.0664375	-3.542482	0.0005929	0.0133609	-0.461895
ATP5IF1	0.1691406	8.1052805	3.5413537	0.0005951	0.0133609	-0.465332
MAN1C1	-0.146238	7.5460413	-3.53101	0.0006163	0.0137765	-0.496783
YWHAQ	-0.225959	11.067693	-3.528077	0.0006224	0.0138539	-0.505689
SF3A3	-0.234849	9.3152043	-3.522599	0.000634	0.0140519	-0.522306
VARS	-0.179858	8.2108546	-3.519137	0.0006415	0.014079	-0.532797
CASP4	0.2098298	11.012855	3.5184583	0.000643	0.014079	-0.534852
BAG3	-0.146344	7.6139552	-3.518245	0.0006434	0.014079	-0.535497
H2AFJ	0.2259102	8.8413125	3.5147417	0.000651	0.0141862	-0.546104
SLC9A8	0.181622	8.3770155	3.5114155	0.0006584	0.0142859	-0.556166
RASSF2	0.231996	12.006245	3.5100865	0.0006613	0.0142901	-0.560185
XPO4	-0.12632	8.2031127	-3.508028	0.0006659	0.0143296	-0.566406
NMT2	-0.214096	8.0819703	-3.504054	0.0006749	0.0144622	-0.578409
CANT1	0.1861428	9.2255727	3.5026732	0.000678	0.0144696	-0.582578
MYC	-0.222673	8.9454936	-3.496424	0.0006924	0.0145878	-0.601424
RRAGD	0.2426138	8.8826999	3.4951768	0.0006953	0.0145878	-0.605184
STOML2	-0.125643	8.673907	-3.494704	0.0006964	0.0145878	-0.606607
EXOSC1	-0.115244	8.9200026	-3.494203	0.0006975	0.0145878	-0.608117
MTURN	0.3338533	9.8827297	3.4941791	0.0006976	0.0145878	-0.60819
WDR54	-0.17114	8.862012	-3.489136	0.0007095	0.0147768	-0.623373
INPP5A	0.1493475	8.2437807	3.4836977	0.0007225	0.0149881	-0.639727
LYST	0.2176611	9.2811592	3.474454	0.0007452	0.0152943	-0.667479
TNFAIP8L1	-0.202864	7.7386244	-3.474161	0.0007459	0.0152943	-0.668356
ANXA2	-0.210733	10.079774	-3.474078	0.0007461	0.0152943	-0.668606
TMEM167A	0.1154287	6.8966024	3.4706237	0.0007548	0.0154108	-0.678961

TOB1	-0.149878	8.0534995	-3.467547	0.0007626	0.0154322	-0.688177
SERTAD2	-0.160168	9.7592405	-3.466734	0.0007646	0.0154322	-0.690612
ENO1	-0.187003	11.897224	-3.466688	0.0007648	0.0154322	-0.69075
ZBTB8OS	0.1673689	7.3439409	3.4643641	0.0007707	0.0154919	-0.697705
FBXO11	0.1283863	9.6088527	3.4615687	0.0007779	0.0155765	-0.706067
RAD17	-0.070401	7.0606716	-3.459135	0.0007843	0.0156429	-0.713342
ZNF438	0.1800795	7.7457116	3.4546059	0.0007962	0.0157023	-0.726872
NOL11	-0.149838	8.6923476	-3.4539	0.000798	0.0157023	-0.72898
RMND5A	0.1155454	6.9869443	3.4533436	0.0007995	0.0157023	-0.73064
RBM4	-0.149344	9.1278431	-3.452078	0.0008029	0.0157023	-0.734417
GLT1D1	0.1178994	7.178971	3.4513351	0.0008049	0.0157023	-0.736634
ST8SIA4	0.1762076	7.5271785	3.4511391	0.0008054	0.0157023	-0.737219
ADA2	-0.178819	9.4015384	-3.443439	0.0008263	0.0160488	-0.760169
PIK3CA	0.1111143	7.4691162	3.4396889	0.0008366	0.0161009	-0.771333
CKAP5	-0.156434	7.8530589	-3.43866	0.0008395	0.0161009	-0.774393
NLRP12	0.2776388	10.181142	3.4382283	0.0008407	0.0161009	-0.775679
RBP7	0.2895652	8.7870294	3.4354097	0.0008486	0.0161009	-0.78406
KLHDC8B	0.2100445	8.075406	3.4352642	0.000849	0.0161009	-0.784492
RALB	0.2198841	11.225962	3.4352442	0.000849	0.0161009	-0.784552
P4HA1	0.1108287	7.7148766	3.4333335	0.0008544	0.0161009	-0.79023
GAR1	-0.111371	7.8786483	-3.433151	0.0008549	0.0161009	-0.790774
KHSRP	-0.129844	7.7265662	-3.432463	0.0008569	0.0161009	-0.792816
LEMD2	-0.099294	8.1345207	-3.428076	0.0008694	0.0162206	-0.805842
BASP1	0.3132579	13.088006	3.4280464	0.0008695	0.0162206	-0.805929
SIGLEC17P	-0.100248	7.0574131	-3.423264	0.0008834	0.0164201	-0.820113
OSBPL11	0.1443148	7.8217327	3.4193869	0.0008948	0.0165594	-0.831601
SRSF10	-0.148506	8.3599236	-3.418508	0.0008974	0.0165594	-0.834204
YOD1	0.4019251	8.5015108	3.4174719	0.0009005	0.0165594	-0.837271
PARP1	-0.216737	9.8250405	-3.414329	0.0009098	0.016673	-0.846573
NGRN	-0.154538	9.8747162	-3.412028	0.0009168	0.0167344	-0.853376
ZDHHC17	0.1855398	8.1722846	3.4110811	0.0009196	0.0167344	-0.856176
MUTYH	-0.118377	7.9488802	-3.407916	0.0009293	0.0167769	-0.865528
ETS1	-0.2727	11.574677	-3.407823	0.0009296	0.0167769	-0.865802
ZFP90	-0.160282	8.55989	-3.407091	0.0009318	0.0167769	-0.867965
PLD3	-0.159253	8.7831149	-3.40533	0.0009373	0.0167769	-0.873165
POLA2	-0.111456	7.5526782	-3.404113	0.000941	0.0167769	-0.876758
TGFBRAP1	-0.173856	7.3225681	-3.403991	0.0009414	0.0167769	-0.877118
FRYL	-0.091571	7.7193487	-3.39651	0.0009649	0.0171362	-0.899176
OPN3	-0.121639	7.3350823	-3.391322	0.0009815	0.0173623	-0.914449
GPR68	-0.151416	7.4589297	-3.390433	0.0009843	0.0173623	-0.917064
BOP1	-0.167116	8.4601396	-3.389411	0.0009876	0.0173623	-0.92007
CR1	0.1854351	7.6179691	3.3878919	0.0009926	0.0173902	-0.924537
PRMT1	-0.164998	9.1794255	-3.380905	0.0010156	0.0177335	-0.945061
POC1B	0.1885553	8.0513483	3.3777982	0.001026	0.0177623	-0.954178
RFC5	-0.086953	7.6730763	-3.377565	0.0010268	0.0177623	-0.954861
PCBP1	-0.12635	12.414814	-3.377343	0.0010275	0.0177623	-0.955512
LETM1	-0.106895	7.3373732	-3.376024	0.001032	0.01778	-0.959381
FKBP3	0.1628824	7.506592	3.3731578	0.0010417	0.0178882	-0.967781
ACOT2	-0.088626	7.1327903	-3.370787	0.0010498	0.017968	-0.974724
CEACAM1	0.3036127	8.7670926	3.3694263	0.0010545	0.0179889	-0.978709
CIRBP	-0.16299	10.597153	-3.365939	0.0010666	0.0181024	-0.988912

PHB	-0.104247	8.1069254	-3.365497	0.0010681	0.0181024	-0.990205
C12orf65	-0.123918	7.560643	-3.359834	0.0010881	0.0183803	-1.006757
TPI1	-0.129952	10.801112	-3.353102	0.0011122	0.0187275	-1.026402
EXOSC2	-0.107685	7.4323369	-3.349289	0.0011261	0.0188587	-1.037515
TNIP1	0.1966306	10.044793	3.3489731	0.0011273	0.0188587	-1.038436
PRIM2	-0.105773	6.9896421	-3.346315	0.0011371	0.0188769	-1.046176
FN3KRP	-0.188297	8.5958717	-3.345812	0.001139	0.0188769	-1.047641
BATF2	0.1888018	7.0610618	3.3457197	0.0011393	0.0188769	-1.047909
DRAM1	0.1679883	7.9704299	3.3441055	0.0011453	0.0189159	-1.052607
CREG1	0.2777914	9.1645676	3.3418457	0.0011538	0.0189745	-1.059181
PDCD10	0.2874867	8.0834952	3.3412027	0.0011562	0.0189745	-1.061051
NUP214	0.2055761	10.82673	3.3348222	0.0011804	0.0192975	-1.079589
MCM7	-0.184979	9.692216	-3.334068	0.0011833	0.0192975	-1.081779
WBP1L	0.1051216	8.1459486	3.3194827	0.0012406	0.0201687	-1.124041
HMGB2	0.2555555	8.4315779	3.3177587	0.0012476	0.0202016	-1.129027
FAM206A	0.1106132	7.6681099	3.3170484	0.0012504	0.0202016	-1.131081
DDHD2	-0.106644	8.2317492	-3.314914	0.0012591	0.0202785	-1.13725
RPS14	-0.184197	12.928319	-3.313726	0.0012639	0.0202935	-1.140682
MRM3	-0.116726	8.0922394	-3.312698	0.0012682	0.0202983	-1.143651
HIRIP3	-0.127574	7.6031754	-3.310577	0.0012769	0.0203665	-1.149774
RPL26L1	0.1974793	7.9507292	3.3097564	0.0012803	0.0203665	-1.152142
ADGRG1	-0.291113	8.6807074	-3.308203	0.0012867	0.0204064	-1.156625
TCEAL9	0.1130255	7.0707323	3.3035326	0.0013063	0.0205754	-1.170089
PPM1B	0.1886386	9.6912766	3.3028193	0.0013093	0.0205754	-1.172144
NAT10	-0.162218	8.3041393	-3.302819	0.0013093	0.0205754	-1.172146
STX3	0.2555874	9.5658263	3.3004964	0.0013191	0.0206675	-1.178834
RPLP0	-0.263716	12.729176	-3.299455	0.0013236	0.0206746	-1.181834
PPM1M	0.1261156	10.006705	3.294921	0.001343	0.020916	-1.194876
LCMT1	-0.103408	8.4083178	-3.286324	0.0013807	0.0214385	-1.219572
LOC100506[illegible]	-0.062983	6.7006982	-3.281298	0.0014032	0.0216466	-1.233983
ATP5PB	-0.163336	9.9131779	-3.280094	0.0014086	0.0216466	-1.237432
MRPS26	-0.11944	7.9153132	-3.279254	0.0014125	0.0216466	-1.239839
WDR4	-0.117109	7.9840629	-3.279004	0.0014136	0.0216466	-1.240554
BCL11B	-0.244997	9.4239703	-3.278693	0.001415	0.0216466	-1.241445
STK3	0.1516746	7.6716242	3.2758435	0.001428	0.0217812	-1.249604
ANXA3	0.4100395	8.181667	3.2738197	0.0014373	0.0218588	-1.255394
RANBP3	-0.092469	8.0818481	-3.269122	0.0014591	0.0221256	-1.268824
WDR6	-0.178496	9.7106519	-3.26489	0.001479	0.0223184	-1.280911
LPAR1	0.1494054	7.2861083	3.2641502	0.0014825	0.0223184	-1.283021
ARHGAP11A	0.1049161	7.114529	3.2631955	0.0014871	0.0223184	-1.285745
YWHAZ	-0.146319	10.315609	-3.262782	0.001489	0.0223184	-1.286926
MCRS1	-0.104482	8.5772232	-3.260648	0.0014992	0.0223845	-1.293011
KLHL22	-0.174398	8.9069086	-3.260056	0.0015021	0.0223845	-1.294698
KPNA4	0.1763179	9.2509127	3.2577357	0.0015133	0.0224283	-1.301312
GPR63	0.0605756	6.6906522	3.2576524	0.0015137	0.0224283	-1.301549
KLHL2	0.2368151	7.9608977	3.2553459	0.0015249	0.0224473	-1.308119
AGO4	0.14754	7.7550248	3.2544957	0.001529	0.0224473	-1.310539
NBEAL2	0.2258868	9.5882305	3.2534887	0.0015339	0.0224473	-1.313406
SCMH1	-0.070837	7.4176819	-3.252945	0.0015366	0.0224473	-1.314954
GFM2	-0.066971	6.8868553	-3.25257	0.0015385	0.0224473	-1.316019
COX10	-0.111305	8.0955334	-3.249823	0.001552	0.0224473	-1.323835

UBE2D4	-0.080825	7.5559236	-3.249524	0.0015535	0.0224473	-1.324684
YIF1A	-0.111646	7.9325416	-3.249521	0.0015535	0.0224473	-1.324694
CCT7	-0.141031	9.7012064	-3.249059	0.0015558	0.0224473	-1.326007
DGCR2	0.1418774	8.73816	3.2485669	0.0015582	0.0224473	-1.327406
EPHX2	-0.156951	7.6384401	-3.247354	0.0015643	0.0224719	-1.330852
TXN	0.2579551	10.942106	3.2438526	0.0015818	0.0226614	-1.340799
TRIM68	-0.092198	7.8099285	-3.238908	0.001607	0.0229578	-1.35483
MGEA5	0.1616583	11.238164	3.2378734	0.0016123	0.0229702	-1.357764
HNRNPUL1	-0.145851	10.352448	-3.229322	0.0016567	0.0235389	-1.381983
ATP1B1	-0.071346	6.9451354	-3.224982	0.0016797	0.0238004	-1.394253
NIP7	-0.140419	8.1297615	-3.223675	0.0016867	0.0238342	-1.397945
TMCC3	0.1528084	7.4016131	3.2218884	0.0016963	0.0239046	-1.402993
CHUK	0.1228739	8.2100952	3.2195319	0.001709	0.0240187	-1.409645
CDYL	-0.067086	7.0375922	-3.210513	0.0017585	0.0245595	-1.435068
GATD3A	-0.106544	8.8710234	-3.210026	0.0017613	0.0245595	-1.436439
IFIT5	0.1971449	7.3608734	3.2099465	0.0017617	0.0245595	-1.436663
NRGN	0.4824445	10.678406	3.2089677	0.0017672	0.0245697	-1.439418
UBL5	0.1771485	8.5882047	3.2067977	0.0017793	0.0246727	-1.445524
REV1	-0.094678	7.7567088	-3.204493	0.0017923	0.0247869	-1.452005
PTBP1	-0.141782	11.331104	-3.201219	0.001811	0.0249781	-1.461206
RNF220	-0.131764	8.4995409	-3.199607	0.0018202	0.025039	-1.465734
RCE1	-0.086233	7.8023866	-3.198022	0.0018294	0.025098	-1.470182
F5	0.2742793	8.1515872	3.192754	0.00186	0.0254512	-1.484959
PTPRC	-0.275895	8.5495553	-3.187573	0.0018906	0.0257418	-1.499472
CSF1R	-0.237038	10.715616	-3.187481	0.0018912	0.0257418	-1.499729
NSDHL	-0.093247	7.539515	-3.184738	0.0019076	0.0258972	-1.507405
RIOK1	-0.071966	7.3634107	-3.181349	0.001928	0.0260718	-1.516882
MDH1	-0.159902	10.270991	-3.180941	0.0019305	0.0260718	-1.518019
GFM1	-0.112364	7.8759063	-3.177102	0.0019539	0.0263199	-1.528743
TLK2	0.0847723	8.2459238	3.1740571	0.0019727	0.0263716	-1.537241
XXYLT1	-0.096717	7.5896648	-3.173808	0.0019743	0.0263716	-1.537934
SLC22A4	0.2555421	8.2725311	3.1733314	0.0019772	0.0263716	-1.539265
PNPO	-0.119624	8.627881	-3.17291	0.0019798	0.0263716	-1.540441
GPATCH1	-0.069399	7.2652856	-3.172369	0.0019832	0.0263716	-1.54195
ARMCX6	-0.095906	7.952643	-3.170621	0.0019941	0.0264488	-1.546821
ZMYND8	-0.085542	7.403847	-3.163686	0.002038	0.0269613	-1.566134
PTGDR	-0.154251	7.480949	-3.162345	0.0020465	0.0270059	-1.569864
PRPS1	-0.142777	8.7367571	-3.160065	0.0020612	0.0270618	-1.576203
SYPL1	-0.127353	8.3695159	-3.160063	0.0020612	0.0270618	-1.576208
MME	0.3752038	10.822752	3.1580341	0.0020743	0.0271655	-1.581846
GTPBP3	-0.087037	7.5934529	-3.155415	0.0020914	0.02732	-1.589119
SLC25A22	-0.119252	7.3571954	-3.151127	0.0021196	0.027606	-1.601017
ZNF362	-0.104928	8.3377637	-3.150475	0.002124	0.027606	-1.602824
ITFG2	-0.130986	8.3200169	-3.145973	0.002154	0.0279269	-1.615299
UBE2A	0.1300064	8.313559	3.1441022	0.0021666	0.0280204	-1.62048
PTPN12	0.2157371	8.973169	3.1391946	0.0022	0.0283121	-1.634055
ECH1	-0.148798	10.130053	-3.138582	0.0022042	0.0283121	-1.635748
WBP11	-0.139135	8.4489254	-3.138389	0.0022056	0.0283121	-1.636281
ATP5MF	0.1310171	8.7224724	3.1370722	0.0022146	0.0283583	-1.639921
GOSR2	-0.084709	7.4397267	-3.135353	0.0022265	0.0284197	-1.644671
PJA2	0.3026412	10.924851	3.1344203	0.002233	0.0284197	-1.647246

MAP2K1	-0.130138	9.0987415	-3.134008	0.0022359	0.0284197	-1.648383
FNDC3A	0.0970673	7.4470377	3.1319918	0.0022499	0.0285287	-1.653949
DDA1	-0.121505	9.0847465	-3.129198	0.0022696	0.0286959	-1.661655
NCKAP1L	-0.143134	8.9824445	-3.128544	0.0022742	0.0286959	-1.663457
RIT1	0.1420491	7.8205001	3.1268617	0.0022861	0.0287668	-1.668094
CMTM6	0.2186132	10.455927	3.1261887	0.0022909	0.0287668	-1.669949
RAB18	0.1081574	7.1858	3.122676	0.0023161	0.0289321	-1.679622
BCL3	0.2273496	9.2352672	3.1221061	0.0023202	0.0289321	-1.681191
RNF216P1	-0.06004	7.2266316	-3.122014	0.0023208	0.0289321	-1.681444
NPIPB9	-0.116688	8.1209666	-3.117969	0.0023501	0.0292273	-1.692569
PCSK7	-0.114377	9.2172693	-3.113498	0.0023829	0.0295644	-1.704855
GPR174	-0.052693	6.7407628	-3.112694	0.0023889	0.0295674	-1.707061
ENOPH1	-0.155587	8.1203914	-3.11129	0.0023993	0.0296256	-1.710917
NUP188	-0.105303	7.3533739	-3.110491	0.0024052	0.0296284	-1.713106
HSPA9	-0.14083	9.1418558	-3.104346	0.0024514	0.0300883	-1.729953
MSTO1	-0.087072	7.2638089	-3.103983	0.0024542	0.0300883	-1.730949
DHRSX	0.1034373	7.2652604	3.1026466	0.0024643	0.0301416	-1.734608
TSTA3	0.2749523	8.1359997	3.1015236	0.0024729	0.0301752	-1.737682
RNF4	-0.122762	8.3839909	-3.098898	0.002493	0.0303496	-1.744866
ARPC4	0.1659221	10.498574	3.0928874	0.0025397	0.030756	-1.761294
CYFIP2	-0.205952	11.371667	-3.092877	0.0025398	0.030756	-1.761321
DNAJC3	0.0951018	7.1367913	3.0913224	0.002552	0.030756	-1.765567
CPD	0.2043009	9.0173834	3.0912227	0.0025528	0.030756	-1.765839
CDA	0.3042416	9.750828	3.0895251	0.0025662	0.030756	-1.770472
TGIF1	-0.093952	7.6602575	-3.089133	0.0025693	0.030756	-1.771542
WDR33	-0.102887	8.8712451	-3.089015	0.0025702	0.030756	-1.771862
FLOT1	0.2214943	9.7011482	3.0885568	0.0025739	0.030756	-1.773113
CBX6	-0.13755	8.9919968	-3.086886	0.0025872	0.0308437	-1.777671
RPS2	-0.117106	14.13216	-3.08317	0.0026169	0.031046	-1.787797
CASP8	-0.139729	7.5194029	-3.081902	0.0026272	0.031046	-1.79125
SLC37A4	-0.107126	7.9186985	-3.081824	0.0026278	0.031046	-1.791463
PAN3	0.1463327	9.6395387	3.0817888	0.0026281	0.031046	-1.791558
TRAF3IP2	-0.10591	8.1377723	-3.077279	0.0026648	0.0313375	-1.803828
CYBB	0.2244403	9.3943972	3.0772725	0.0026648	0.0313375	-1.803847
SGSM1	-0.075181	6.9640085	-3.074728	0.0026858	0.031512	-1.810765
C16orf87	-0.087902	7.112135	-3.073389	0.0026968	0.0315705	-1.814403
KRTCAP2	-0.16019	10.056077	-3.071018	0.0027165	0.0316869	-1.820843
HNRNPA1P1(	-0.253968	10.886934	-3.070723	0.002719	0.0316869	-1.821642
SH2D3C	-0.122725	8.644775	-3.067933	0.0027424	0.031798	-1.829213
ARHGAP26	0.146617	7.4850776	3.0675721	0.0027454	0.031798	-1.830193
KLHL12	0.1020494	8.3399691	3.067392	0.0027469	0.031798	-1.830681
NOP53	-0.157103	13.062847	-3.063748	0.0027778	0.0320094	-1.84056
PPP3CC	-0.150302	8.2825413	-3.062895	0.0027851	0.0320094	-1.842872
CRELD1	-0.095902	7.5596423	-3.06281	0.0027858	0.0320094	-1.843101
ASCC1	-0.10229	8.3326579	-3.061626	0.0027959	0.0320094	-1.846307
ZNF317	-0.07289	7.8472181	-3.061058	0.0028008	0.0320094	-1.847845
GAB2	0.2235907	9.3036766	3.0595359	0.0028139	0.0320094	-1.851966
AHSA1	-0.10748	8.6538719	-3.058609	0.0028218	0.0320094	-1.854474
NOA1	-0.108508	8.8526163	-3.058535	0.0028225	0.0320094	-1.854674
DSE	0.1848356	8.4594717	3.0582339	0.0028251	0.0320094	-1.855489
RBBP4	-0.14596	7.7636242	-3.058025	0.0028269	0.0320094	-1.856055

UBAC2	-0.124558	8.7217689	-3.056559	0.0028396	0.0320368	-1.860021
MIS18BP1	0.1641321	8.972411	3.0549015	0.002854	0.0320368	-1.864501
NFIL3	0.2184997	8.7221572	3.0542462	0.0028598	0.0320368	-1.866273
ANK3	-0.062151	6.8865212	-3.054234	0.0028599	0.0320368	-1.866305
KIR2DL3	-0.3669	8.5747717	-3.054091	0.0028611	0.0320368	-1.866692
PYHIN1	-0.208667	8.5891469	-3.053491	0.0028664	0.0320368	-1.868315
PHF19	-0.110077	7.9427054	-3.051938	0.00288	0.03212	-1.872509
PELI1	0.2516227	9.2772145	3.048873	0.0029071	0.0323526	-1.880784
QDPR	-0.114376	8.2585334	-3.046754	0.002926	0.0324606	-1.886501
KLHL35	0.0799721	7.0114111	3.046201	0.0029309	0.0324606	-1.887992
RPS16	-0.132286	13.892147	-3.045679	0.0029356	0.0324606	-1.889398
WDR43	-0.07374	7.1733127	-3.043383	0.0029562	0.0325552	-1.895589
WTAP	0.1153251	8.2773479	3.0433311	0.0029567	0.0325552	-1.895728
THBS1	0.1655271	7.2118582	3.0416149	0.0029722	0.0326567	-1.900351
CALM3	-0.158236	11.348182	-3.037606	0.0030088	0.0329095	-1.911144
PANX2	0.2733286	8.8557187	3.0372598	0.0030119	0.0329095	-1.912074
DET1	-0.06096	7.2581796	-3.035817	0.0030252	0.0329095	-1.915954
FAIM	-0.072093	6.9191971	-3.035714	0.0030262	0.0329095	-1.916231
LONP2	-0.112231	8.1824525	-3.035438	0.0030287	0.0329095	-1.916973
ARHGEF7	-0.085479	8.1003892	-3.034939	0.0030333	0.0329095	-1.918316
MYPOP	-0.078946	8.7786551	-3.033654	0.0030452	0.0329695	-1.921769
EPHB1	0.1270694	7.4567001	3.0327277	0.0030538	0.0329937	-1.924259
AP4B1	-0.122064	8.842794	-3.03125	0.0030676	0.0330735	-1.928229
DHPS	-0.091857	9.014308	-3.027399	0.0031037	0.0333839	-1.938566
ZDHHC14	-0.086744	7.2193951	-3.026813	0.0031092	0.0333839	-1.940137
AIM2	0.2598404	8.4666798	3.0255779	0.0031209	0.03339	-1.94345
ROCK2	0.0940241	11.086421	3.0253927	0.0031227	0.03339	-1.943946
ZNF271P	0.1007685	8.2010922	3.024227	0.0031337	0.0334395	-1.947072
IL37	0.1357262	7.4621249	3.0178126	0.0031953	0.0340268	-1.96425
MAP1LC3B	0.133085	9.4494893	3.0163932	0.0032091	0.0340452	-1.968048
RTN3	0.1542254	8.832389	3.0162835	0.0032102	0.0340452	-1.968341
SERPINB1	0.1913017	10.328202	3.0145533	0.0032271	0.0341543	-1.972968
DKC1	-0.117313	9.1516169	-3.008638	0.0032854	0.034693	-1.988768
TCTN3	-0.083482	7.3440074	-3.007871	0.003293	0.034693	-1.990817
PPP1R3B	0.0894414	7.0043075	3.0073701	0.003298	0.034693	-1.992152
PDCL	-0.076314	6.9335245	-3.004268	0.0033291	0.0348982	-2.000426
RAB24	0.1968992	10.946564	3.004081	0.003331	0.0348982	-2.000924
KLHL36	-0.108788	7.8986725	-3.001545	0.0033566	0.0350958	-2.007681
DENND2D	-0.17586	10.06022	-2.999557	0.0033769	0.0352362	-2.012975
ZFYVE16	0.1149771	7.2713414	2.9961343	0.0034119	0.0355306	-2.022083
SLC6A6	0.1253429	7.4254367	2.9939409	0.0034346	0.0356949	-2.027915
EAF1	0.091422	7.6916424	2.9903423	0.003472	0.0359439	-2.037476
GPKOW	-0.102021	7.88582	-2.990308	0.0034724	0.0359439	-2.037566
NAMPT	0.3062305	11.093914	2.9868493	0.0035088	0.0362353	-2.046747
LSS	-0.121175	7.7753079	-2.986304	0.0035145	0.0362353	-2.048192
TTC5	-0.122705	7.8125257	-2.984543	0.0035332	0.0363557	-2.052864
RABL2A	-0.074307	7.1517062	-2.983845	0.0035406	0.03636	-2.054712
MSRB2	0.2135007	9.5656124	2.9825383	0.0035546	0.0363682	-2.058177
LPAR2	0.2518788	9.9947745	2.9824565	0.0035555	0.0363682	-2.058393
PCCA	-0.073241	7.4064629	-2.975337	0.0036324	0.0370817	-2.077239
UBE2R2	0.0716039	7.0733024	2.9722472	0.0036662	0.0373537	-2.085405

PDLIM2	-0.049584	6.6832061	-2.970992	0.0036801	0.0374107	-2.088722
TCP1	-0.176292	8.236565	-2.970432	0.0036862	0.0374107	-2.090201
WDR61	-0.126084	8.6407072	-2.969187	0.0037	0.0374772	-2.093485
PDLIM7	0.1973735	8.7857704	2.9683461	0.0037094	0.0374987	-2.095706
ATP2A3	-0.122106	8.1530333	-2.96629	0.0037323	0.0376571	-2.101131
BRD9	-0.11492	9.3815465	-2.964226	0.0037555	0.0378171	-2.106574
RPLP1	-0.178475	12.943921	-2.962463	0.0037753	0.0378539	-2.11122
PTPN9	-0.058347	6.9639506	-2.962177	0.0037786	0.0378539	-2.111974
ALDH1A1	-0.199896	8.156359	-2.961962	0.003781	0.0378539	-2.11254
APOBEC3A	0.3241692	8.2713867	2.9598349	0.0038052	0.0380222	-2.118141
CSTF2	-0.092714	7.7624307	-2.95732	0.0038339	0.0382356	-2.124759
HDLBP	0.0515337	6.887029	2.9529313	0.0038845	0.0386662	-2.136298
ATP5MC2	-0.140666	11.187387	-2.950882	0.0039084	0.0387537	-2.141682
ZC3H15	0.1302417	7.4509596	2.9506088	0.0039115	0.0387537	-2.142399
CNIH4	0.2298242	9.1761738	2.9499219	0.0039196	0.0387537	-2.144203
NUP85	-0.07843	8.495278	-2.949296	0.0039269	0.0387537	-2.145845
VPS8	0.1361361	8.9430239	2.9489757	0.0039307	0.0387537	-2.146686
FRY	0.0847351	7.0457889	2.9465236	0.0039595	0.0389642	-2.15312
TGOLN2	-0.165239	10.012408	-2.941348	0.0040211	0.039495	-2.166684
ABLIM1	-0.159618	8.8213406	-2.93842	0.0040563	0.0397423	-2.174349
WDR18	-0.143089	8.6340899	-2.937983	0.0040616	0.0397423	-2.175493
CYB5B	-0.121312	9.9621204	-2.934382	0.0041053	0.0400948	-2.184909
STAU1	0.1398955	9.6935304	2.9318095	0.0041369	0.0402023	-2.191631
KMT5B	0.1083198	8.2514728	2.9316792	0.0041385	0.0402023	-2.191971
SLC2A14	0.1257252	7.251147	2.9315872	0.0041396	0.0402023	-2.192212
PLPPR2	0.1961575	8.320735	2.9299332	0.00416	0.0402596	-2.19653
NHS	0.0935806	7.0979133	2.9298492	0.004161	0.0402596	-2.196749
LCMT2	-0.094892	7.4857326	-2.928629	0.0041762	0.0403306	-2.199934
TRAPPC6B	0.140536	7.3182876	2.9274793	0.0041904	0.0403396	-2.202934
ANP32A-IT	0.1697926	8.6009502	2.9267272	0.0041998	0.0403396	-2.204895
RTN2	0.1100548	7.3476695	2.9266518	0.0042007	0.0403396	-2.205092
SLC6A13	0.0516432	6.9077507	2.9260545	0.0042082	0.0403396	-2.206649
LAMTOR3	0.1939515	9.2560374	2.9236974	0.0042377	0.0405478	-2.212793
C21orf91	0.0656953	7.2138613	2.9221189	0.0042576	0.0406631	-2.216905
ZAP70	-0.157181	8.4777744	-2.920874	0.0042734	0.0407385	-2.220147
SKAP2	0.2254991	9.5244615	2.9200939	0.0042833	0.0407579	-2.222178
PCED1B	-0.190106	9.8616929	-2.917441	0.0043171	0.0408101	-2.229082
SLF2	-0.093167	8.1929491	-2.91699	0.0043229	0.0408101	-2.230254
DGAT2	0.1824324	7.9225727	2.9166789	0.0043268	0.0408101	-2.231063
CCAR2	-0.10322	7.5996468	-2.916164	0.0043334	0.0408101	-2.232401
PTPN23	-0.086535	7.0803329	-2.915574	0.004341	0.0408101	-2.233936
LY6H	0.0796384	7.26643	2.9146167	0.0043533	0.0408101	-2.236424
PTGS2	0.2301382	8.3270693	2.9142205	0.0043584	0.0408101	-2.237453
ACOT1	-0.058087	6.9101985	-2.913959	0.0043618	0.0408101	-2.238134
PHC2	0.1764448	8.1855514	2.9131884	0.0043718	0.0408101	-2.240135
MFSD5	-0.093413	8.6529444	-2.912667	0.0043785	0.0408101	-2.24149
RAB20	0.1115132	7.0871825	2.9124862	0.0043809	0.0408101	-2.241959
MAT2B	-0.184102	9.3595324	-2.912	0.0043872	0.0408101	-2.243222
CIB1	-0.15598	11.268577	-2.911702	0.0043911	0.0408101	-2.243996
RAPH1	-0.047952	6.7646126	-2.909695	0.0044172	0.0408194	-2.249206
IL27RA	-0.168236	9.3866937	-2.909368	0.0044215	0.0408194	-2.250055

NFKB1	-0.130531	11.270764	-2.909337	0.0044219	0.0408194	-2.250134
RPS6KA3	0.139985	7.5867174	2.9092088	0.0044235	0.0408194	-2.250467
LINC01184	-0.079406	7.0808347	-2.905508	0.0044722	0.0411897	-2.260064
MED10	-0.105757	8.5972844	-2.904951	0.0044796	0.0411897	-2.261508
TBCD	-0.126273	8.0204521	-2.897456	0.0045797	0.0420364	-2.280912
LRIG1	-0.109591	7.5606829	-2.894883	0.0046146	0.0422645	-2.287562
RING1	-0.121799	9.7682216	-2.894421	0.0046209	0.0422645	-2.288757
ENDOD1	0.1601429	7.6317436	2.8921755	0.0046516	0.04247	-2.294556
NOP2	-0.123109	8.1301026	-2.889886	0.004683	0.0426273	-2.300466
ACAA2	-0.10416	7.944466	-2.889726	0.0046852	0.0426273	-2.300879
ALG11	-0.061319	6.9891627	-2.889106	0.0046938	0.0426302	-2.302476
TFPI	0.0960776	6.9035508	2.8883379	0.0047044	0.042652	-2.304459
ZMYM2	0.0918547	7.7446675	2.8872915	0.0047189	0.0427089	-2.307157
EIF2B5	-0.10543	7.8078873	-2.885496	0.0047439	0.0428601	-2.311785
RHEX	0.0885245	6.9257423	2.8825618	0.004785	0.043144	-2.319343
C3orf62	0.1327504	7.9315907	2.8820662	0.0047919	0.043144	-2.320619
LFNG	-0.159154	11.067631	-2.879576	0.0048271	0.0433463	-2.327028
PSMC4	-0.170377	7.8724849	-2.878882	0.0048369	0.0433463	-2.328812
TMA7	0.2956395	10.624817	2.8784701	0.0048428	0.0433463	-2.329872
HMGN2	-0.12318	11.298814	-2.877813	0.0048521	0.0433463	-2.331562
EIF4G1	-0.120076	7.9904904	-2.877042	0.0048631	0.0433463	-2.333545
NAGPA	-0.096013	8.223327	-2.876943	0.0048645	0.0433463	-2.333798
HSD17B7	0.2147744	12.549853	2.8760038	0.004878	0.0433913	-2.336212
HMGN1	-0.181609	10.722208	-2.87315	0.004919	0.0435933	-2.343543
PINK1	0.2151417	9.0312548	2.8727467	0.0049248	0.0435933	-2.344577
SAMD3	-0.216377	8.9884525	-2.872669	0.0049259	0.0435933	-2.344776
DDX24	-0.165042	8.9786387	-2.871061	0.0049492	0.0437246	-2.348904
EIF2B4	-0.103913	9.1449011	-2.868335	0.0049888	0.0440001	-2.355897
PLAC8	-0.205442	10.749719	-2.865699	0.0050275	0.0442655	-2.362651
KIDINS220	0.1545694	8.7309435	2.8638527	0.0050547	0.0443541	-2.367381
DFFA	-0.119528	7.9540194	-2.863372	0.0050618	0.0443541	-2.368611
APEX2	-0.085263	8.0497913	-2.863279	0.0050632	0.0443541	-2.36885
GLO1	-0.145706	9.0411286	-2.862269	0.0050782	0.0444103	-2.371435
USP8	0.085407	7.9338457	2.8608174	0.0050997	0.0444586	-2.375149
FAM46C	0.4924792	11.37646	2.8607445	0.0051008	0.0444586	-2.375336
RPL13A	-0.239474	11.40833	-2.857115	0.0051552	0.0448571	-2.384616
TRIM21	0.1721262	9.2378432	2.8532276	0.005214	0.0452929	-2.394543
GNB2	0.1371328	8.3065897	2.8517234	0.0052369	0.045416	-2.398382
CBX3	0.1749421	8.2638914	2.8501627	0.0052608	0.045506	-2.402363
ABHD5	0.2135804	9.1348153	2.8498986	0.0052649	0.045506	-2.403036
IRAK3	0.2305921	8.3890591	2.8482415	0.0052904	0.0456502	-2.407261
NRG1	0.1459862	6.9884174	2.8470086	0.0053094	0.0457383	-2.410403
ZBTB9	-0.091323	7.7013938	-2.845144	0.0053383	0.0459111	-2.415151
AXIN1	-0.097466	8.459152	-2.843577	0.0053627	0.0460446	-2.41914
LARP7	0.1472809	7.7190313	2.8420315	0.0053869	0.0461757	-2.423073
ZNF792	-0.075112	7.4697688	-2.840587	0.0054095	0.0462359	-2.426747
LIN37	0.0600426	7.1850879	2.8404486	0.0054117	0.0462359	-2.427099
ZW10	-0.088772	7.8408232	-2.839068	0.0054335	0.0463455	-2.430609
TRIM32	-0.074323	7.2744967	-2.836964	0.0054668	0.0465532	-2.435955
ATN1	-0.077798	6.8148501	-2.836087	0.0054808	0.0465955	-2.438183
ZNF823	-0.065441	7.1938017	-2.833801	0.0055173	0.0468291	-2.443987

LAGE3	-0.133336	9.0330749	-2.832733	0.0055344	0.0468355	-2.446695
RUFY2	0.0366025	6.856304	2.8316471	0.0055519	0.0468355	-2.44945
SPON2	-0.149293	7.1759091	-2.8313	0.0055575	0.0468355	-2.450331
C2orf42	-0.078611	8.2929788	-2.831136	0.0055601	0.0468355	-2.450747
EIF3F	-0.163955	11.190506	-2.830945	0.0055632	0.0468355	-2.45123
STXBP5	0.1404433	8.1976022	2.8290594	0.0055937	0.0469857	-2.456009
PANK2	0.0922297	9.6452984	2.8281771	0.005608	0.0469857	-2.458245
IRAK4	0.0979497	7.7692849	2.8281655	0.0056082	0.0469857	-2.458274
RASSF1	-0.113407	8.5005214	-2.826356	0.0056377	0.0471566	-2.462858
MTMR14	-0.117097	9.7176048	-2.821906	0.0057108	0.0476206	-2.474118
KANSL2	-0.125436	8.8130075	-2.821863	0.0057115	0.0476206	-2.474225
AHDC1	-0.092572	6.9879003	-2.820651	0.0057316	0.0477112	-2.477289
SLC25A37	0.3171376	12.650214	2.8193035	0.005754	0.0478083	-2.480695
ZNHIT6	-0.118794	7.8913845	-2.818841	0.0057617	0.0478083	-2.481863
MATK	-0.164404	8.3147558	-2.812949	0.0058607	0.0485518	-2.496736
XPNPEP1	-0.105492	9.3954725	-2.811192	0.0058905	0.0486948	-2.501165
SERPING1	0.2861731	7.5247442	2.8108252	0.0058967	0.0486948	-2.502089
TTC26	0.0708683	6.9247837	2.8100948	0.0059092	0.04872	-2.503929
RANGAP1	-0.166832	8.9935123	-2.809311	0.0059225	0.0487528	-2.505904
TTN	-0.056404	6.7901217	-2.806648	0.0059682	0.0490289	-2.512609
SERPINB6	-0.135295	10.218058	-2.805815	0.0059826	0.0490289	-2.514705
ATP5MC1	-0.119795	9.0536926	-2.805181	0.0059935	0.0490289	-2.5163
C20orf27	-0.1393	8.2858265	-2.805159	0.0059939	0.0490289	-2.516356
ALMS1	-0.092102	7.2216597	-2.803473	0.0060231	0.0491901	-2.520595
GMDS	-0.10083	8.1360907	-2.802631	0.0060377	0.049232	-2.522712
EIF3A	-0.111261	8.5772267	-2.801996	0.0060488	0.0492448	-2.52431
TAF1	-0.060964	6.9469605	-2.798614	0.006108	0.0496487	-2.532805
HIC2	-0.087186	7.5883749	-2.797003	0.0061363	0.0497713	-2.53685
C5AR1	0.2663466	10.643631	2.7964594	0.0061459	0.0497713	-2.538213
EHMT2	-0.066189	6.9615864	-2.796124	0.0061519	0.0497713	-2.539055
CXCR1	0.2838329	10.07267	2.795584	0.0061614	0.0497713	-2.540409
